# VCP and ATL1 regulate endoplasmic reticulum and protein synthesis for dendritic spine formation

**DOI:** 10.1038/ncomms11020

**Published:** 2016-03-17

**Authors:** Yu-Tzu Shih, Yi-Ping Hsueh

**Affiliations:** 1 Molecular Cell Biology, Taiwan International Graduate Program, Institute of Molecular Biology, Academia Sinica, and Graduate Institute of Life Sciences, National Defense Medical Center, Taipei 115, Taiwan; 2 Institute of Molecular Biology, Academia Sinica, 128, Academia Road Sec 2, Nankang, Taipei 115, Taiwan

## Abstract

Imbalanced protein homeostasis, such as excessive protein synthesis and protein aggregation, is a pathogenic hallmark of a range of neurological disorders. Here, using expression of mutant proteins, a knockdown approach and disease mutation knockin mice, we show that VCP (valosin-containing protein), together with its cofactor P47 and the endoplasmic reticulum (ER) morphology regulator ATL1 (Atlastin-1), regulates tubular ER formation and influences the efficiency of protein synthesis to control dendritic spine formation in neurons. Strengthening the significance of protein synthesis in dendritic spinogenesis, the translation blocker cyclohexamide and the mTOR inhibitor rapamycin reduce dendritic spine density, while a leucine supplement that increases protein synthesis ameliorates the dendritic spine defects caused by *Vcp* and *Atl1* deficiencies. Because *VCP* and *ATL1* are the causative genes of several neurodegenerative and neurodevelopmental disorders, we suggest that impaired ER formation and inefficient protein synthesis are significant in the pathogenesis of multiple neurological disorders.

## Abstract (web summary)

Protein homeostasis is crucial for maintaining a variety of cellular functions. Here the authors show that valosin-containing protein and its cofactors regulate tubular ER formation and protein synthesis efficiency, thereby control dendritic spine formation in neurons.

## Graphical abstract

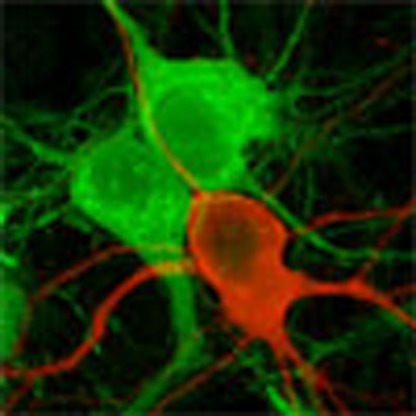

Protein homeostasis, an integrated outcome of protein synthesis and degradation, is crucial for the maintenance of a variety of cellular functions. Imbalanced protein homeostasis caused by excessive protein synthesis, defective protein degradation and protein aggregation is known to be associated with many neurological disorders^1^,^2^,^3^,^4^,^5^. *VCP*, also known as *P97*, is one of the genes controlling protein homeostasis and is also associated with neurological disorders.

*Vcp* encodes a hexameric AAA+ ATPase that functions as a chaperone to control diverse cellular processes, including endoplasmic reticulum (ER)-associated protein degradation (ERAD)^6^,^7^, the ubiquitin–proteasome system (UPS)^8^,^9^, ER and Golgi morphogenesis^10^,^11^,^12^,^13^,^14^,^15^,^16^,^17^ and others^9^,^18^,^19^. The binding partners of VCP determine its diverse activities^9^,^20^,^21^. For example, when the ubiquitin fusion degradation 1-like (UFD1L)–nuclear protein localization homolog 4 (NPL4) heterodimer binds VCP, the VCP complex is involved in ERAD and UPS^6^,^7^,^9^ (Fig. 1a, bottom). While VCP interacts with another cofactor named P47, also known as the NSFL1 (P97) cofactor, it regulates membrane fusion in ER and Golgi morphogenesis^10^,^11^,^12^,^13^,^14^,^22^ (Fig. 1a, top). P47 competes with the UFD1L–NPL4 heterodimer to interact with the N-terminal region of VCP^23^. Thus, it is expected that the expression levels of the cofactors influence the function of VCP in cells by competing for interactions and targeting VCP to different protein machineries.

VCP proteins are ubiquitously expressed in various tissues. Mutations in the *VCP* gene results in multisystem disorders, such as inclusion body myopathy associated with Paget's disease of bone and frontotemporal dementia (IBMPFD)^24^, amyotrophic lateral sclerosis^25^,^26^ and autism spectrum disorders^27^. Neurological dysfunction is shared among these diseases. Our previous study showed that VCP interacts with another disease molecule, neurofibromin, which is encoded by the *Neurofibromatosis type I* gene, and acts downstream of neurofibromin to regulate dendritic spine formation—a subcellular location of excitatory synapses^28^. The involvement of VCP in dendritic spine formation provides a potential explanation for the dysregulation of neuronal function in patients with *VCP* mutations, although it is unclear how VCP controls dendritic spine formation.

Because the function of VCP is determined by its cofactor, in this report, we investigate the functions of two major VCP cofactors—P47 and the UFD1L-NPL4 dimer—to explore how VCP controls dendritic spinogenesis. Our results show that P47, but not the UFD1L-NPL4 dimer, is involved in VCP-mediated dendritic spine formation. Our study suggests that the VCP-P47 complex acts with an ER regulator, ATL1, to regulate ER morphology and protein synthesis, which are critical for dendritic spinogenesis.

## Results

### P47 acts with VCP to regulate dendritic spine density

**Figure 1:**
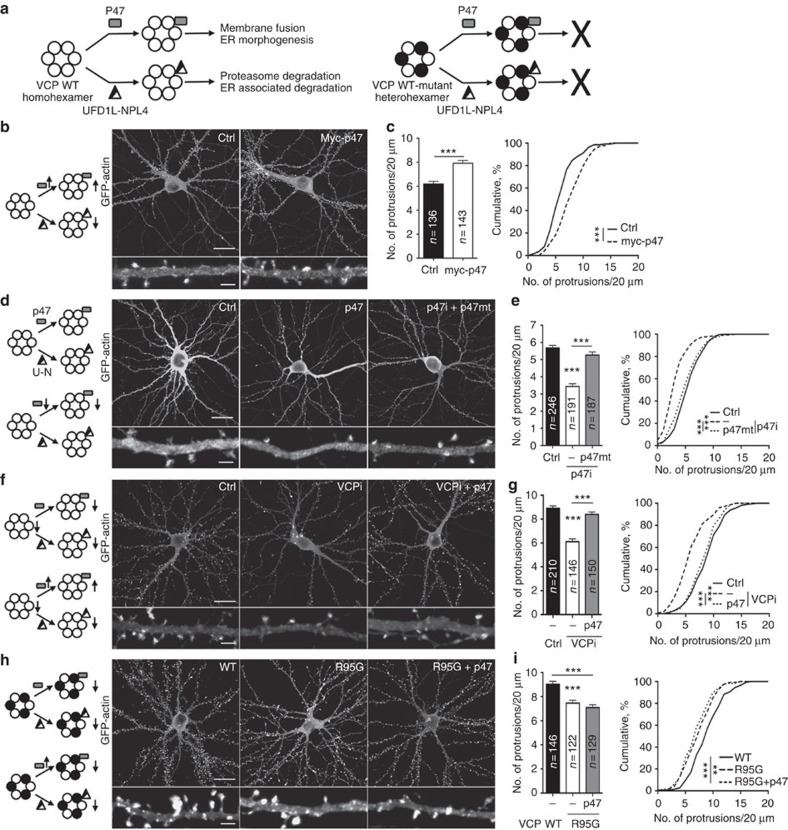
P47 acts downstream of VCP to regulate dendritic spine formation. (**a**) A schematic of VCP hexamers and their cofactors, P47 and the UFDL1-NPL4 dimer, in two distinct functions. Heterohexamers containing WT (open circle) and mutant (closed circle) VCP are inactive. (**b**,**d**,**f**,**h**) Cultured neurons were cotransfected with GFP-actin and the indicated plasmids at 12 days *in vitro* (DIV) and fixed for dendritic spine analysis at 18 DIV. The expression of the various constructs was monitored by immunofluorescence staining, though only the GFP signals are shown to reveal the neuronal morphology. The left panels illustrate the experimental design and the predicted activity of the VCP complexes. Arrows pointing up or down indicate either the changes in the protein levels or the activities of the complexes. In the images, the lower panel enlargements are the quantitated segments from the upper panels. (**c**,**e**,**g**,**i**) Quantitation of the protrusion densities collected from three independent experiments. The means plus s.e.m. and the cumulative probability are shown. The sample sizes (*n*) of the analyzed dendrites are indicated. Ctrl, non-silencing control. Scale bars: original, 20 μm; enlarged, 2 μm. **P<0.01; ***P<0.001. Unpaired *t*-test (**c**); one-way ANOVA (**e**,**g**,**h**); Kolmogorov–Smirnov test for cumulative probability (**c**,**e**,**g**,**i**).

In our previous proteomic study, P47—the cofactor guiding VCP-mediated regulation of ER membrane fusion—formed a complex with VCP and neurofibromin in rat brain extracts^28^. So we investigated whether P47 is also involved in dendritic spinogenesis. Cultured hippocampal neurons were transfected with *P47* at 12 days *in vitro* (DIV) and then we analyzed dendritic spine density at 18 DIV. We found that compared with a vector control, neurons transfected with *P47* had a higher density of dendritic spines (Fig. 1b,c). To further confirm the function of P47, we applied an RNA interference approach to reduce P47 expression. A *P47* RNAi knockdown construct (P47i) that reduced *P47* expression in both transfected COS-1 cells and cultured hippocampal neurons (Supplementary Fig. 1a,b) was transfected into cultured neurons. GFP signals from the knockdown vector and coexpressed GFP-actin were used to indicate transfected cells and also to outline the neuronal morphology and dendritic spines. Similar to previous results for *Vcp* and *Nf1* deficiencies^28^,^29^, the reduction of *P47* expression decreased the density of dendritic spines (Fig. 1d,e). Coexpression of the *P47* silent mutant that is resistant to P47i (Supplementary Fig. 1a) rescued the dendritic spine defects caused by *P47* knockdown (Fig. 1d,e), suggesting the specificity of P47i. To minimize personal bias, we conducted blind morphometry experiments for this report.

We then investigated the involvement of P47 in VCP-mediated dendritic spinogenesis. Both knockdown of endogenous VCP and overexpression of the VCP R95G mutant—an IBMPFD mutant—were shown to reduce the number of dendritic spines^28^. In *Vcp* knockdown neurons (∼60% reduction^28^), *P47* overexpression likely recruits the residual VCP to perform P47-dependent activities and may ameliorate the spine defects. In contrast, expression of the VCP R95G mutant impairs the activities of all hexameric VCP in neurons (Fig. 1a, right). Therefore, P47 overexpression was not expected to rescue the defect caused by the VCP R95G mutant (Fig. 1f,h, left panels). Indeed, P47 overexpression increased the dendritic spine density in *Vcp* knockdown neurons (Fig. 1f,g), but not in the neurons expressing the VCP R95G mutant (Fig. 1h,i). The results suggest that VCP uses a P47-dependent pathway to regulate dendritic spine density.

### UFD1L-dependent protein degradation is not critical

In addition to the P47-dependent pathway, we also investigated whether protein degradation controlled by the VCP–UFD1L–NPL4 complex (Supplementary Fig. 2a) is involved in dendritic spine formation. Because UFD1L and NPL4 function as a dimer, a reduction of either one is sufficient to impair their function. We knocked down *Ufd1l* expression in COS-1 cells and cultured hippocampal neurons (Supplementary Fig. 2b,c) and found that the dendritic spine density was not altered (Supplementary Fig. 2d,e). To ensure that *Ufd1l* knockdown by Ufd1i impairs protein degradation, we used an ERAD reporter (CD3δ-GFP) and an unstable fluorescence protein (Ub^G76V^-GFP) to monitor protein degradation. These GFP fusion proteins are unstable and rapidly degraded. An accumulation of GFP signal therefore indicates impaired protein degradation. Compared with the non-silencing control, Ufd1i expression induced clear punctate signals for both CD3δ-GFP and Ub^G76V^-GFP (Supplementary Fig. 2f,g), suggesting that *Ufd1l* knockdown promoted unfolded protein accumulation. To further confirm that unfolded protein accumulation does not impair dendritic spinogenesis, we treated the cultured neurons with tunicamycin, which blocks the synthesis of N-linked glycoproteins, to induce unfolded protein accumulation and ER stress. Tunicamycin treatment induced eIF2 phosphorylation and CD3δ-GFP accumulation (Supplementary Fig. 3a,b). We also labeled newly-synthesized proteins for 1 h with the methionine analog L-azidohomoalanine (AHA) at 18 DIV using a well-established bioorthogonal non-canonical amino acid tagging (BONCAT) method^30^. After washing, AHA-labeled proteins were detected using tetramethylrhodamine (TAMRA)-conjugated alkyne combined with TAMRA antibody. Notably, protein synthesis was transiently inhibited at 2 and 6 h after tunicamycin treatment, but then gradually recovered (Supplementary Fig. 3c). These analyses indicated that tunicamycin induced an unfolded protein response and led to protein accumulation in neurons. However, tunicamycin treatment did not alter the dendritic spine density and cell morphology of neurons (Supplementary Fig. 3d). Echoing the lack of effect of tunicamycin on dendritic spine formation, the expression levels of synaptic proteins—such as N-methyl-D-aspartate receptor subunits GRIN2A and GRIN2B (also known as NMDAR2A and NMDAR2B), *α*-amino-3-hydroxy-5-methyl-4-isoxazolepropionic acid receptor subunit GRIA2/3 (also known as GluR2/3), metabotrophic glutamate receptor GRM5 (also known as mGluR5) and synaptic scaffold proteins PSD-95 and CASK—did not exhibit a noticeable reduction pattern after tunicamycin treatment (Supplementary Fig. 3e). Perhaps, these synaptic proteins are relatively stable^31^ and cannot be influenced by transient suppression of protein translation. Thus, although tunicamycin treatment induced an unfolded protein response, it had a very limited effect on dendritic spines.

Together, these results suggest that an unfolded protein response is unlikely to be involved in VCP-dependent dendritic spinogenesis.

### VCP and P47 regulate dendritic ER distribution in neurons

**Figure 2:**
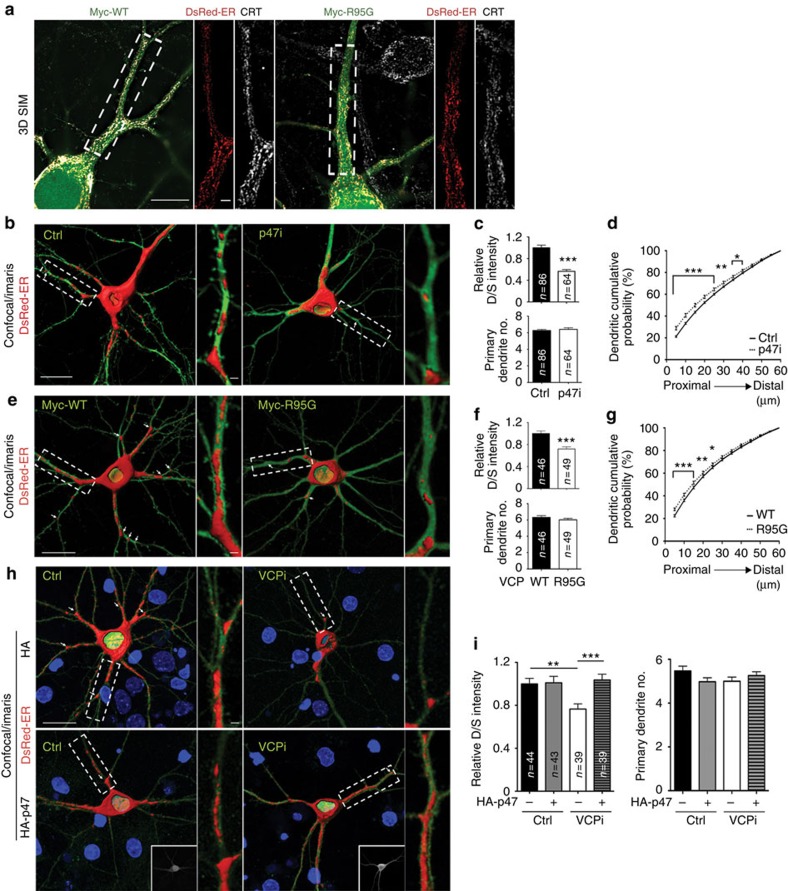
VCP and P47 regulate dendritic ER distribution. (**a**) 3D SIM images of cultured hippocampal neurons. DsRed-ER and Myc-tagged WT *Vcp* or the R95G mutant were cotransfected into neurons at 12 DIV and analyzed with immunostaining using Myc, DsRed and the endogenous ER marker calreticulin (CRT) antibodies at 18 DIV. (**b**–**i**) DsRed-ER was cotransfected with the indicated plasmids into cultured neurons. The Z-series of the confocal images were processed with the Surpass Mode of the Imaris software (Bitplane) as shown in (**b**,**e**,**h**). Arrows identify the examples of the dendritic branch sites with enriched ER. (**c**,**f**,**i**) The ratio of dendritic DsRed to somatic DsRed (D/S) and the total number of dendrites. (**d**,**g**) The cumulative probability of dendritic DsRed-ER shows the accumulation of DsRed-ER in the proximal region of the dendrites in P47i- and R95G-expressing cells. Ctrl, non-silencing control. Scale bars: (**a**) original, 10 μm; enlarged, 2 μm; (**b**,**e**,**h**) original, 20 μm; enlarged, 2 μm. The data are from three independent experiments and are presented as the mean plus s.e.m. (error bars). The sample sizes (*n*) of the examined neurons are indicated. *P<0.05; **P<0.01; ***P<0.001. Unpaired *t*-test (**c**,**f**); two-way ANOVA (**d**,**g**,**i**).

In dividing cells, the VCP–P47 complex is well known to control the homotypic membrane fusion of intracellular organelles, including the ER^12^,^13^,^15^,^16^,^17^. However, the role of the VCP–P47 complex in ER morphogenesis and its subsequent physiological function is completely unknown in neurons. Because local tubular ER complexity in dendrites is correlated with the density of local dendritic spines^32^, we investigated whether the VCP–P47 protein complex regulates tubular ER formation and ER extension into the dendrites. DsRed-ER—a red fluorescent protein (DsRed) fused with the ER targeting sequence of calreticulin at its N-terminal end and the ER retention sequence KDEL at its C-terminal end—was used to label the ER in cultured neurons. The pattern of DsRed-ER in neurons overlapped well with that of endogenous calreticulin, as analyzed by 3D-SIM (Fig. 2a), supporting that DsRed-ER signals reflect the endogenous ER distribution. Similar to a previous finding^32^, DsRed-ER was enriched at the dendritic branch points and the bases of dendritic spines (Fig. 2a,b,e,h).

The ratio of dendritic DsRed-ER to somatic DsRed-ER (D/S ratio) was then determined to investigate whether the *Vcp* and *P47* deficiencies regulate tubular ER extension in neurons. The D/S ratio of *P47* knockdown neurons was considerably lower than that of control neurons (Fig. 2b,c). Because *P47* knockdown did not noticeably change the number of dendrites (Fig. 2c, bottom panel), the lower D/S ratio suggested that less ER was distributed into the individual dendrites in the *P47* knockdown neurons. When we measured the cumulative probability of DsRed-ER along the dendrites, a relatively higher percentage of ER was present in the proximal dendrites of *P47* knockdown neurons (Fig. 2d), indicating a deficit in ER extension along dendrites. Similar ER defects were also found in VCP R95G mutant-expressing and *Vcp* knockdown neurons (Fig. 2e–i). These results suggest that *Vcp* and *P47* deficiencies reduce ER extension from the somata into the dendrites.

For dendritic spinogenesis, *P47* overexpression effectively ameliorated the dendritic spine defect caused by *Vcp* knockdown (Fig. 1f,g). If the ER defects are relevant to the dendritic spine defects, we expected that *P47* overexpression would rescue the ER defects caused by *Vcp* knockdown. Indeed, overexpression of *P47* in the *Vcp* knockdown neurons increased the D/S ratio of DsRed-ER to the level of control neurons (Fig. 2h–i). Thus, VCP and P47 work together to control ER distribution and dendritic spine formation.

### VCP and P47 regulate ER distribution *in vivo*

**Figure 3:**
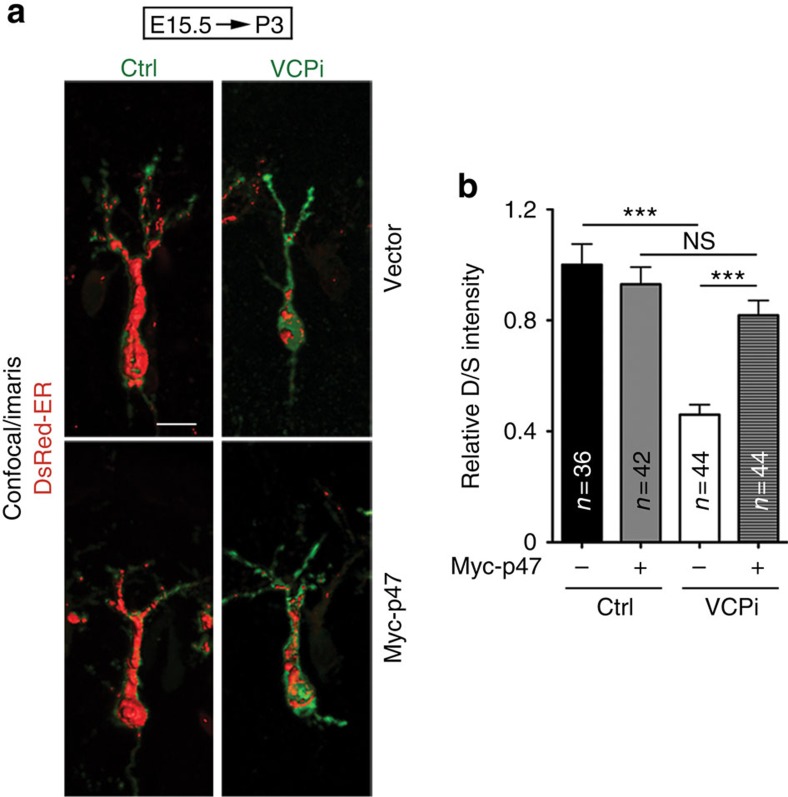
*Vcp* knockdown *in vivo* impairs ER distribution in neurons. (**a**) In utero electroporation with the indicated plasmids was performed at E15.5 of CD1 mice. Layer 2/3 cortical neurons were then immunostained with GFP, DsRed and Myc antibodies at P3 to outline the cell morphology and determine the ER distribution in the neurons. The Z-series of the confocal images were processed with the Surpass Mode of the Imaris software (Bitplane). (**b**) The ratio of dendritic DsRed to somatic DsRed (D/S). Scale bar: 10 μm. Ctrl, non-silencing control. Four to five pups for each group were analyzed. The numbers of the examined neurons (*n*) are indicated. The data are presented as the mean+s.e.m. (error bars). NS, non-significant; ***P<0.001; two-way ANOVA.

In addition to cultured neurons, we further evaluated the role of VCP and P47 in ER distribution *in vivo* using in utero electroporated mice. VCPi and DsRed-ER were delivered into the cerebral cortex at embryonic day 15.5. Layer 2/3 cortical neurons were then analyzed at postnatal day 3. Consistent with the results from the cultured neurons, the D/S ratio of DsRed-ER was noticeably reduced in the *Vcp* knockdown neurons (Fig. 3a,b). This suggests that ER extension into the dendrites was also impaired in layer 2/3 cortical neurons when the expression levels of *Vcp* were reduced *in vivo*. In addition, *P47* overexpression ameliorated the ER extension defects caused by *Vcp* knockdown (Fig. 3a,b). Taken together, both the *in vivo* and *in vitro* studies suggest that the VCP–P47 complex regulates tubular ER formation and extension in neurons.

### Somatic rough ER is also impaired by the VCP R95G mutation

**Figure 4:**
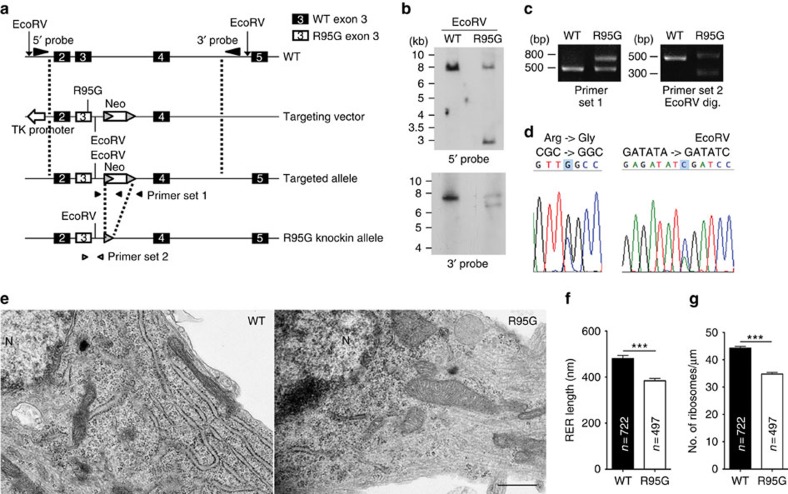
VCP R95G knockin mutant neurons have altered rough ER in somata. (**a**) Schematic of mouse *Vcp* genomic organization and design of the R95G knockin-targeting construct. Dark box and open box indicates WT and *Vcp*^*R95G*^ (R95G) mutated exons, respectively. (**b**) Genomic Southern blotting analysis with the 5′ probe: 7.1 kb for WT and additional 2.7 kb for *Vcp*^*wt/R95G*^ (R95G) mice; 3′ probe: 7.1 kb for WT and additional 6.2 kb for R95G mice. (**c**) Genotyping by PCR with primer set 1: products of 475 bp for WT and additional 711 bp for R95G mice; primer set 2: products of 497 bp for WT and 500 bp for R95G allele. The R95G PCR product was digested with EcoRV to generate 236 and 264 bp fragments. In (**b**,**c**), images have been cropped for presentation. Full-size images are presented in Supplementary Fig. 9. (**d**) Sequencing results of the PCR product amplified by primer set 2 to reveal R95G and EcoRV mutations. (**e**) TEM analysis of cultured hippocampal neurons from WT and R95G mice at 18 DIV. Neurons were collected from nine mice for each group from three independent experiments. Number of analyzed neurons: WT, 120; R95G, 132. Error bars present the mean+s.e.m.. N, nucleus. (**f**,**g**) The length and ribosomal density of rER were quantified. Scale bar: 500 nm. ***P<0.001; unpaired *t*-test.

Because the ER is a continuous membrane structure extending from the nuclear envelope to the entire cell, the dendritic ER defect caused by *Vcp* and *P47* deficiencies (Figs 2 and 3) suggests that the ER in soma is also likely to be affected. To investigate this possibility, we generated mutant mice expressing VCP R95G mutant proteins using a gene targeting approach (Fig. 4a–d) and cultured hippocampal neurons from mutant embryos and wild-type littermates. Because IBMPFD is dominantly inherited, *Vcp*^*WT/R95G*^ mice (referred to as VCP R95G knockin mice hereafter) were used to examine the effect of the *Vcp* mutation. At 18 DIV, cultured neurons were analyzed using transmission electron microscopy (TEM) (Fig. 4e). The length of individual rough ER (rER) and the density of attached ribosomes on ER were determined. We found that the rER length was noticeably reduced in VCP R95G knockin neurons (Fig. 4e,f). Note that we collected blind a similar number of images from VCP R95G knockin neurons and wild-type neurons and found that rER fragments in VCP R95G knockin neurons were much fewer. This led to a lower sample size of rER in VCP R95G knockin neurons (Fig. 4f,g, wild-type, *n*=722; R95G, *n*=497). These data suggest that the VCP R95G mutation reduced rER extension and abundance. Moreover, we found that the density of ribosomes attached to ER was also much lower in VCP R95G knockin neurons (Fig. 4e,g), which should influence protein synthesis activity in VCP R95G knockin neurons. Together, the results from TEM suggest that the VCP R95G mutation impairs ER morphology and function in soma.

### Protein synthesis is impaired by *Vcp* and *P47* deficiency

**Figure 5:**
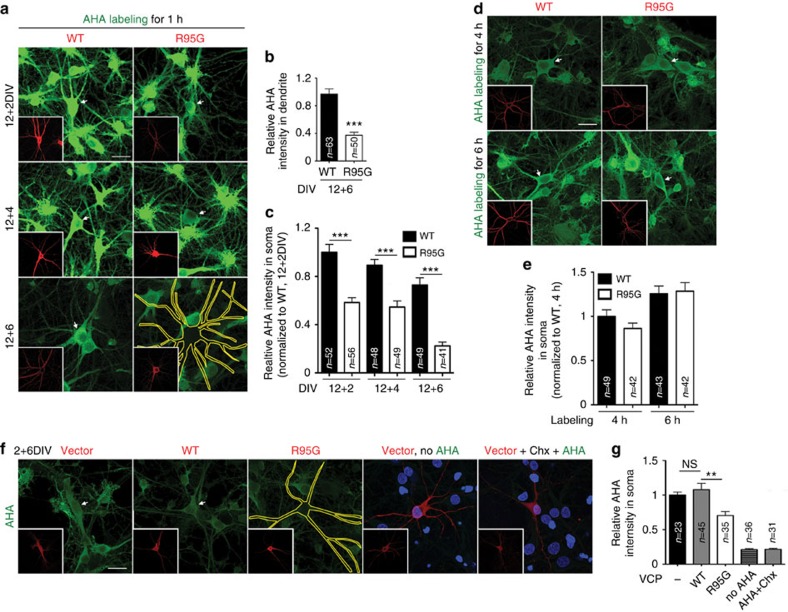
Expression of the VCP R95G mutant reduces protein synthesis efficiency. Cultured neurons were transfected with WT VCP or the R95G mutant at (**a**–**e**) 12 DIV and (**f**,**g**) 2 DIV, and protein synthesis was analyzed using AHA labeling at 2, 4 or 6 days later, as indicated. AHA labeling was detected with alkyne-modified Alexa Fluor-488. (**a**,**d**,**f**) Representative images of the AHA labeling. The insets indicate the transfected cells, which are either outlined or indicated by arrows in the original images. (**b**) Relative AHA intensities in the dendrites. (**c**,**e**,**g**) The relative AHA signals in the somata. In (**a**–**c**,**f**,**g**), AHA was labeled for 1 h; in (**d**,**e**), AHA was added to the culture for 4 or 6 h, as indicated. In (**f**), AHA omission or cycloheximide addition (Chx; 10 μM) was used to confirm the specificity of the AHA labeling. Scale bar: 25 μm. The sample sizes (*n*) of the analyzed neurons are indicated. The data from three independent experiments are presented as the mean+s.e.m. (error bars). NS, non-significant; **P<0.01; ***P<0.001. Unpaired *t*-test (**b**,**c**,**e**); one-way ANOVA (**g**).

Because abundance of and ribosomal attachment to rER was noticeably affected by *Vcp* deficiency and because the major function of rER is to synthesize proteins, including both membrane/secreted proteins and cytosolic proteins^33^,^34^,^35^, we investigated the effects of VCP and P47 on protein synthesis. Newly-synthesized proteins were labeled by a BONCAT method^30^. After extensive washing to remove free AHA, AHA-incorporated proteins were then detected by Alexa Fluor-488-conjugated alkyne. We found that the expression of the VCP R95G mutant inhibited protein synthesis in dendrites (Fig. 5a,b). Furthermore, protein synthesis in somata was also noticeably reduced (Fig. 5a,c), suggesting a global defect in protein synthesis in VCP R95G mutant neurons. This global reduction of protein synthesis was also shown in cultured hippocampal neurons at 18 DIV prepared from VCP R95G knockin mice (Supplementary Fig. 4a,b). Expression of the VCP R95G mutant in cultured neurons exhibited time-dependent effects. Expression for 6 days (DIV 12+6) reduced newly-synthesized protein by an average of 70% (Fig. 5a,c), while neurons expressing the R95G mutant for 2 (DIV 12+2) or 4 days (DIV 12+4) inhibited protein synthesis by ∼40% (Fig. 5a,c). In addition, when AHA labeling was extended to 4 or 6 h, there was no obvious difference between the AHA signals in the WT- and R95G mutant-expressing neurons (Fig. 5d,e). The lack of a difference after the longer incubation time is not likely to be due to saturation of AHA labeling because the AHA signals are stronger with longer labeling time (Fig. 5d,e). These data suggest that *Vcp* deficiency impairs the efficiency of protein synthesis in neurons.

In addition to mature neurons, we also examined the effect of the VCP R95G mutant in immature neurons. Cultured hippocampal neurons were transfected with WT VCP or the R95G mutant at 2 DIV and were analyzed at 8 DIV. Similar to the results using mature neurons at 18 DIV, expression of the VCP R95G mutant also reduced protein synthesis in the immature neurons at 8 DIV (Fig. 5f,g). The controls for omitting AHA and adding cycloheximide showed no obvious signal, supporting the specificity of AHA (Fig. 5f,g). Consistent with the defects in protein synthesis, the ER distribution in the dendrites was also reduced in the VCP R95G mutant-expressing neurons at 8 DIV (Supplementary Fig. 5a,b). Together, these results suggest that *Vcp* deficiency impairs both ER distribution and protein synthesis in immature as well as mature neurons.

**Figure 6:**
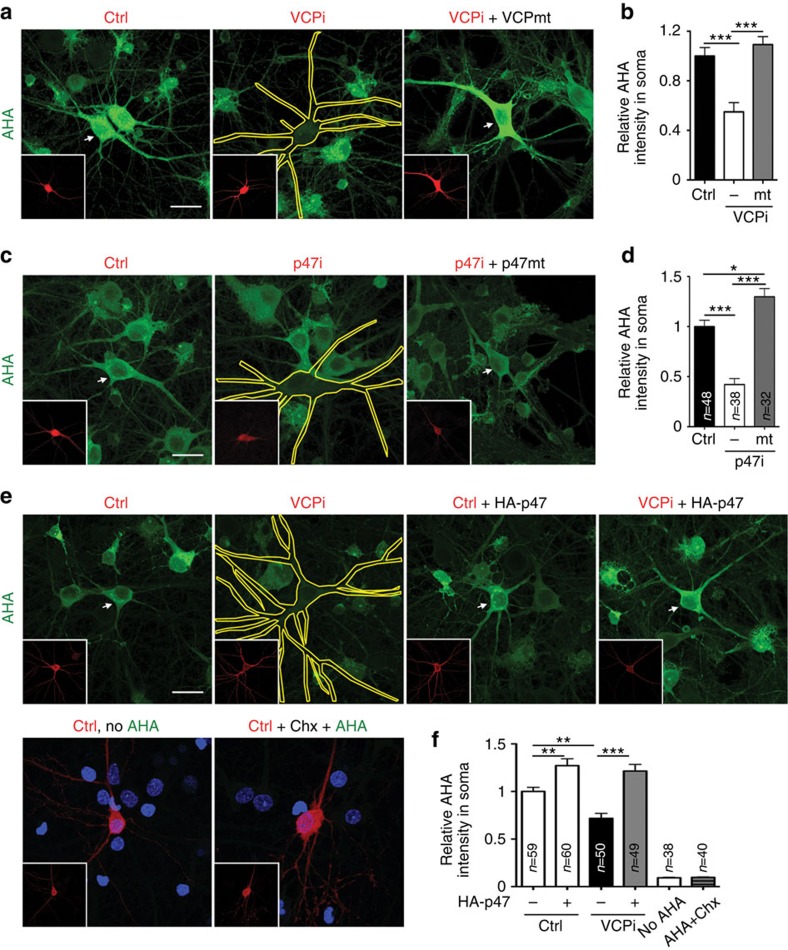
*Vcp* and *P47* knockdown impair protein synthesis. Cultured neurons were cotransfected with the indicated knockdown and expression constructs at 12 DIV and subjected to AHA labeling for 1 h at 18 DIV. (**a**,**c**,**e**) Representative images of AHA labeling. The insets indicate the transfected cells, which are outlined or indicated by arrows in the original images. (**b**,**d**,**f**) The relative AHA intensity in somata. In (**e**), AHA omission or cycloheximide addition (Chx; 10 μM) was used to confirm the specificity of the AHA labeling. To indicate the presence of neurons, DAPI signals are shown in these two groups. Ctrl, non-silencing control. Scale bar: 25 μm. The sample sizes (*n*) of the examined neurons are indicated. The data from three independent experiments are presented as the mean+s.e.m. (error bars). *P<0.05; **P<0.01; ***P<0.001. One-way ANOVA (**b**,**d**), two-way ANOVA (**f**).

Knockdown of *Vcp* and *P47* also had a negative effect on protein synthesis (Fig. 6a–d). The negative effects were specifically due to the reduction of *Vcp* and *P47* expression because the expression of *Vcp* and *P47* silent mutants that are resistant to the *Vcp* and *P47* knockdown constructs rescued protein synthesis in neurons expressing the respective knockdown constructs (Fig. 6a–d). Moreover, similar to the ER distribution and dendritic spine formation, overexpression of *P47* effectively increased protein synthesis in *Vcp* knockdown neurons (Fig. 6e,f). Thus, VCP and P47 work together to regulate ER distribution, protein synthesis and dendritic spine formation in neurons.

In addition to BONCAT, we applied another method—surface sensing of translation (SUnSET)^36^—to monitor protein synthesis through the incorporation of puromycin into neurons. Puromycin resembles the 3′ end of transfer RNA and incorporates into synthesizing polypeptide chains^37^. Puromycin-labeled proteins can then be detected using puromycin antibody. Similar to the results using AHA labeling, *Vcp* knockdown reduced puromycin labeling and *P47* overexpression rescued puromycin incorporation in *Vcp* knockdown neurons (Supplementary Fig. 6). In conclusion, both BONCAT and SUnSET reveal the protein synthesis defects caused by *Vcp* and *P47* deficiencies.

### Some synaptic ion channels are less expressed in *Vcp* mutants

**Figure 7:**
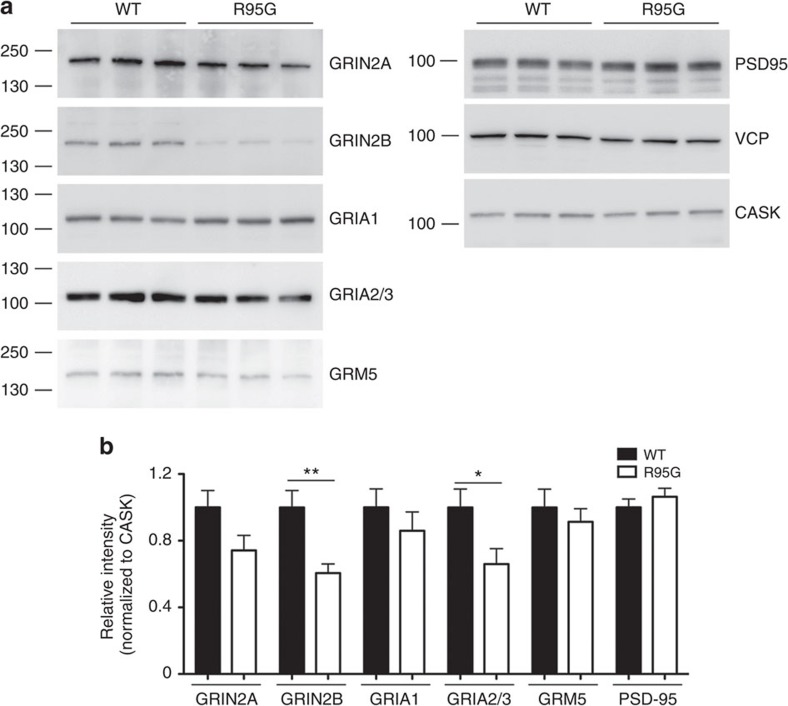
Expression of synaptic membrane proteins GRIN2B and GRIA2/3 are reduced in VCP R95G knockin mutant neurons. (**a**) Synaptic protein expressions of hippocampal neurons from WT and VCP R95G mice at 18 DIV. (**b**) Quantification of (**a**), normalized with CASK. Extracts collected from a total of six different mice for each genotype are compared. The data are presented as the mean+s.e.m. (error bars). *P<0.05; **P<0.01; unpaired *t*-test. Images have been cropped for presentation. Full-size images are presented in Supplementary Fig. 9.

To more closely examine its control over dendritic spine density, in addition to its effect on protein synthesis revealed by AHA and puromycin labeling, we also wondered whether *Vcp* deficiency influences the expression of synaptic membrane proteins. We screened several glutamate receptors, which are enriched at dendritic spines and are critical for their functioning, and found that expression levels of GRIN2B and GRIA2/3 were noticeably lower in VCP R95G knockin neurons (Fig. 7a,b). GRIN2A levels also tended to be lower in VCP R95G knockin neurons, but the difference did not attain statistical significance. For GRIA1 and GRM5, their protein levels were comparable between WT and VCP R95G knockin neurons (Fig. 7a,b). PSD-95 protein levels were also unaffected by the VCP R95G mutation (Fig. 7a,b). These results suggest that some synaptic ion channels are particularly sensitive to the VCP R95G mutation.

### Inhibition of protein synthesis reduces synapse density

**Figure 8:**
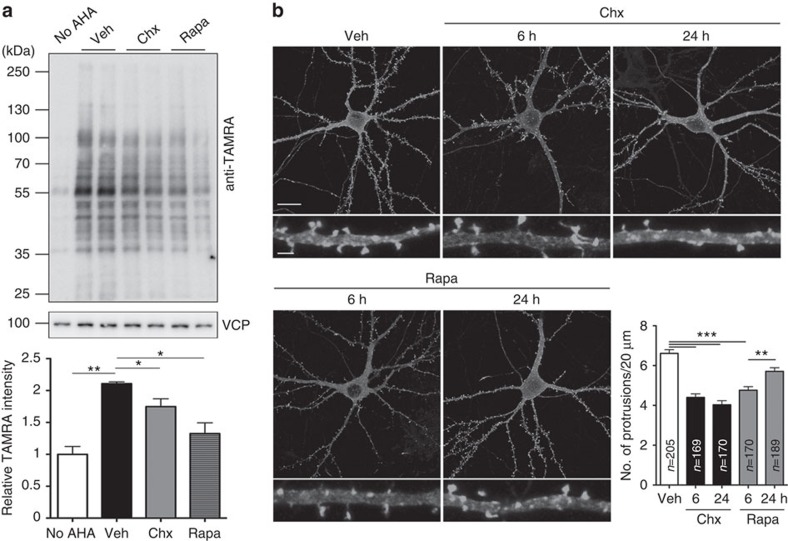
Inhibition of translation using cycloheximide and rapamycin reduces the dendritic spine density. (**a**) Cycloheximide (Chx, 10 μM) and rapamycin (Rapa, 10 nM) treatment for 6 h inhibited protein synthesis of cultured neurons as revealed by the AHA signals with the TAMRA antibody. Images have been cropped for presentation. Full-size images are presented in Supplementary Fig. 9. (**b**) Cultured neurons transfected with GFP-actin to outline dendritic spines were treated with Chx and Rapa for 6 or 24 h before immunostaining with the GFP antibody. Veh, vehicle. Scale bars: original, 20 μm; enlarged, 2 μm. The sample sizes (*n*) of the examined dendrites are indicated. The data from three independent experiments are presented as the mean+s.e.m. (error bars). *P<0.05; **P<0.01; ***P<0.001; one-way ANOVA.

The aforementioned results imply that defective protein synthesis likely reduces the dendritic spine density in *Vcp*- and *P47*-deficient neurons. Under this scenario, we expected that inhibition of protein synthesis with other treatments should also reduce dendritic spine density. Cycloheximide and rapamycin—an inhibitor of the mammalian target of rapamycin (mTOR)^38^,^39^—were used to investigate this possibility. As expected, preincubation with cycloheximide and rapamycin reduced AHA incorporation in neurons (Fig. 8a). More importantly, cycloheximide and rapamycin treatment also reduced the dendritic spine density (Fig. 8b). The inhibitory effects of cycloheximide and rapamycin on the dendritic spine density are consistent with our hypothesis that protein synthesis is critical for the regulation of dendritic spine density.

### Restoring protein synthesis ameliorates the synapse defects

**Figure 9:**
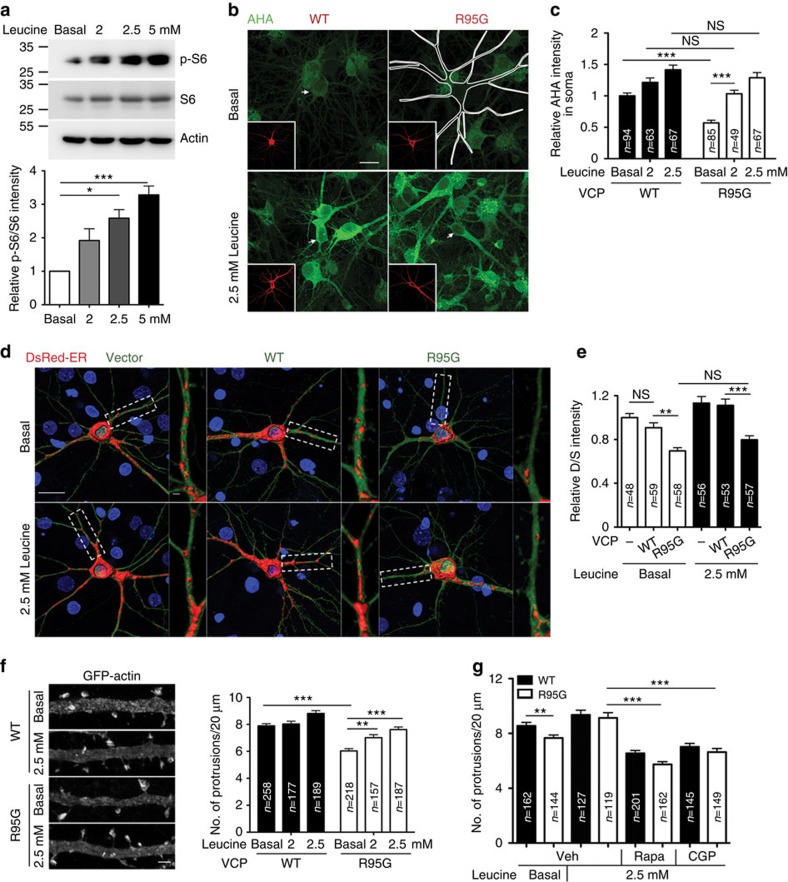
Leucine supplementation ameliorates the deficits in protein synthesis and dendritic spine density of *Vcp*-deficient neurons. (**a**) Increased leucine concentrations in the medium induce the phosphorylation of S6 ribosomal proteins in the cultured neurons. The basal leucine concentration in Neurobasal medium is 0.8 mM. Full-size blots are shown in Supplementary Fig. 9. (**b**–**g**) Cultured neurons were transfected with the indicated plasmids at 12 DIV, treated with different concentrations of leucine at 15 DIV and analyzed for (**b**,**c**) AHA incorporation, (**d**,**e**) ER distribution and (**f**,**g**) dendritic spine density at 18 DIV. Full-size cell images of (**f**) are shown in Supplementary Fig. 7a. In (**b**), the transfected neurons in the images are either outlined or indicated by arrows. (**g**) Rapamycin (Rapa; 10 nM) and MNK1 inhibitor CGP57380 (CGP; 10 μM) were added 6 h before harvesting to reduce the beneficial effect of leucine on dendritic spine density. Full-size cell images of (**g**) are shown in Supplementary Fig. 7b. Scale bars, (**b**,**d**) 20 μm; (**d**), enlarged and (**f**) 2 μm. The data from three independent experiments are presented as the mean+s.e.m. (error bars). The sample sizes (*n*) of the examined neurons (**c**,**e**) and dendrites (**f**,**g**) are indicated. *P<0.05; **P<0.01; ***P<0.001; NS, non-significant. One-way ANOVA (**a**); two-way ANOVA (**c**,**e**,**f**,**g**).

If protein synthesis is indeed the key downstream pathway of VCP and P47 that controls dendritic spine formation, upregulation of protein synthesis in *Vcp*- and *P47*-deficient neurons would be expected to ameliorate the dendritic spine defects. Leucine, a branched-chain amino acid, is known to increase protein synthesis by activating the mTOR pathway^40^,^41^,^42^. Additional leucine was added into culture media to increase concentrations from the basal level (0.8 mM) to 2, 2.5 or 5 mM. We found that the additional leucine effectively induced S6 ribosomal protein phosphorylation in neurons in a dose-dependent manner (Fig. 9a). The addition of leucine to 2.5 mM in cultured neurons was sufficient to ameliorate the defects in protein synthesis caused by the VCP R95G mutant (Fig. 9b,c). However, the additional leucine did not improve ER distribution in VCP R95G mutant neurons (Fig. 9d,e). More importantly, we found that the additional leucine increased the dendritic spine density of the VCP R95G mutant-expressing neurons to the level of the WT VCP-expressing neurons (Fig. 9f). To further confirm the role of protein synthesis in the effect of leucine on the dendritic spine density, rapamycin and CGP57380—an inhibitor of MAP kinase-interacting serine/threonine-protein kinase 1 (MNK1)^43^—were applied to neurons treated with additional leucine. We found that both inhibitors effectively suppressed the effect of the additional leucine on dendritic spine formation (Fig. 9g and Supplementary Fig. 7a,b).

Taken together, these results suggest that leucine is able to ameliorate the defects in dendritic spines caused by a *Vcp* deficiency by inducing protein synthesis. These results strengthen our hypothesis that inefficient protein synthesis is the critical impairment downstream of *Vcp* deficiency in controlling dendritic spine formation.

### ATL1 and RAB10 control protein synthesis and synapse density

**Figure 10:**
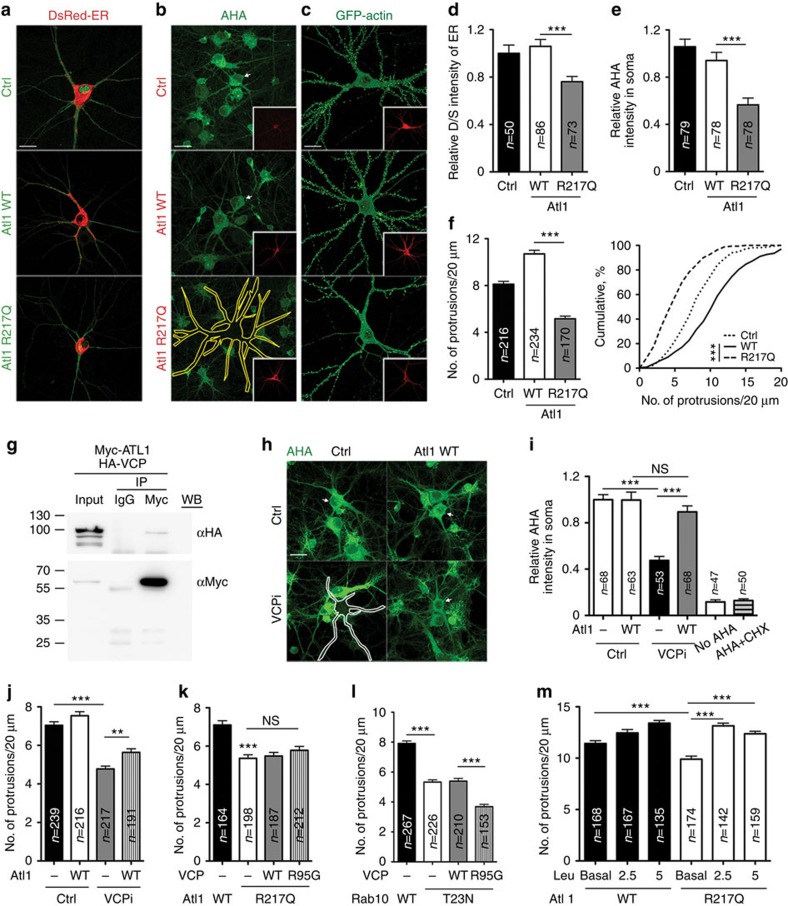
ATL1 and VCP act together in regulating dendritic spine formation. (**a**-**c**,**h**) Rat cultured hippocampal neurons were transfected with plasmids, as indicated, and subjected to analyses of (**a**) ER morphology, (**b**,**h**) protein synthesis based on AHA labeling and (**c**) neuronal morphology using GFP signals. Transfected neurons are indicated either by arrowheads or yellow outline. ER signals in (**a**) were processed with the Surpass Mode of the Imaris software (Bitplane). (**d**) Quantification of the D/S ratio of DsRed-ER. (**e**,**i**) Relative intensities of AHA-Alexa fluor 488 in soma. (**f**) Quantitation of dendritic protrusion densities. (**g**) Co-immunoprecipiation of VCP and ATL1 from transfected COS-1 cells. Full-size images are available in Supplementary Fig. 9. (**j**–**m**) GFP-actin was cotransfected with various plasmids into cultured neurons as indicated to analyze the density of dendritic spines. (**j**) *Atl1* expression partially rescued the effect of *Vcp* knockdown. (**k**) The ATL1 R217Q mutant and VCP R95G mutant did not have an additive effect on dendritic spine density. (**l**) Expression of the RAB10 T23N mutant further reduced the density of dendritic spines in VCP R95G-expressing cells. (**m**) Extra leucine increased the dendritic spine density of ATL1 R217Q mutant-expressing neurons. Scale bar: (**a**–**c**,**h**) 20 μm. The data from three independent experiments are presented as the mean+s.e.m. (error bars). Cumulative probability distributions of spine density are also shown. The sample sizes (*n*) are indicated. **P<0.01; ***P<0.001; NS, non-significant. One-way ANOVA (**d**,**e**,**f**); two-way ANOVA (**i**–**m**).

To further investigate the involvement of ER malformation and dysfunction in dendritic spinogenesis, we examined the function of ATL1 and RAB10 in cultured hippocampal neurons. *ATL1* is a causative gene of hereditary spastic paraplegia 3A (SPG3A) and encodes a dynamin-like GTPase to control homotypic membrane fusion of tubular ER^44^,^45^,^46^. The R217Q mutation of *ATL1* with defective GTPase activity has been identified in patients with SPG3A^47^. Cultured hippocampal neurons were cotransfected with DsRed-ER and the WT or R217Q mutant of ATL1. As expected, ATL1 R217Q mutant-expressing cells had a lower D/S ratio of DsRed-ER compared with neurons transfected with WT *Atl1* or control vector (Fig. 10a,d). Consistent with our hypothesis, expression of the ATL1 R217Q mutant noticeably impaired protein synthesis, which was reflected in lower AHA incorporation (Fig. 10b,e), and a reduced density of dendritic spines (Fig. 10c,f). This reduction was specifically caused by the *Atl1* mutation because expression of WT *Atl1* did not impair ER distribution or protein synthesis. In fact, we even found an increase in dendritic spine density for ATL1 WT (Fig. 10a–f). Thus, these results suggest a role for ATL1 in the regulation of ER distribution, protein synthesis and dendritic spinogenesis.

The small GTPase RAB10 also regulates tubular ER formation. However, it is involved in ER tubule growth independently of ATL1 (ref. 48). To examine whether RAB10 also influences protein synthesis and dendritic spine formation, the effects of WT and a GDP-locked T23N mutant of RAB10 in cultured hippocampal neurons were compared. The RAB10 T23N mutant had been shown to inhibit the function of endogenous RAB10 in tubular ER formation^48^. As expected, inhibition of endogenous RAB10 activity by expression of the RAB10 T23N mutant impaired the ER distribution in neurons (Supplementary Fig. 8a,d). Although the mechanisms underlying the function of ATL1 and RAB10 in ER formation differ^48^, we found that similar to ATL1, the RAB10 T23N mutant also reduced protein synthesis and dendritic spine formation (Supplementary Fig. 8b,c,e,f). Together, the ATL1 and RAB10 results echo our hypothesis that regulation of the tubular ER network controls protein synthesis and consequently influences the density of dendritic spines.

### VCP and ATL1 act together for dendritic spine formation

A recent study indicated that, in Drosophila, VCP functionally interacts with ATL1 (ref. 49). It seems very likely that VCP and ATL1 also work together in mammalian neurons to regulate dendritic spinogenesis. To examine this possibility, we first confirmed the interaction between VCP and ATL1 in mammalian cells by co-immunoprecipitation (Fig. 10g). The rescue effect of overexpression of *Atl1* on *Vcp* knockdown was then investigated. We found that *Atl1* overexpression increased protein synthesis and partially rescued the density of dendritic spines in *Vcp* knockdown neurons (Fig. 10h–j). Expression of both VCP R95G and ATL1 R217Q mutants in cultured neurons did not have an additive effect on downregulation of dendritic spine formation (Fig. 10k). These results suggest that VCP and ATL1 act in the same pathway to control dendritic spine formation. In contrast to ATL1, expression of the RAB10 T23N mutant further decreased the dendritic spine density in the presence of the VCP R95G mutant (Fig. 10l). These results lend further credence to our hypothesis that VCP and ATL1, but not RAB10, work in the same pathway to regulate dendritic spine formation.

To investigate the involvement of protein synthesis in the effect of ATL1 on dendritic spine formation, extra leucine was added to neurons expressing the ATL1 R217Q mutant. Similar to *Vcp*-deficient neurons, extra leucine also ameliorated the dendritic spine defect caused by the ATL1 R217Q mutant (Fig. 10m).

Since ATL1 is a well-known regulator for tubular ER formation, the physical and functional interactions between VCP and ATL1 further support that VCP controls tubular ER formation to regulate dendritic spine formation.

## Discussion

VCP is involved in multiple cellular processes. The current model of the pathogenic mechanism of VCP-related disorders mainly focuses on protein degradation involving UPS, ERAD and autophagy^9^,^50^. One of the reasons why protein degradation is particularly interesting is because protein aggregation in patient tissues has been recognized as a hallmark of VCP-related disorders^24^,^51^. However, the evidence does not exclude the involvement of other functions of VCP in the pathogenic mechanism(s) of diseases. Previous studies have suggested that the influence of *VCP* mutations on different types of cells varies. In mouse myoblast C2C12 cells, expression of the *VCP* IBMPFD mutants induces the aggregation of polyubiquitinated proteins^52^. In contrast, although the *VCP* IBMPFD mutant proteins do not form obvious aggregates in cultured hippocampal neurons after expression for six days, dendritic spine density was reduced^28^, suggesting that protein aggregation is not required for the *VCP* IBMPFD mutant-mediated impairment of dendritic spinogenesis. These findings imply that the pathogenic mechanisms underlying the *VCP* defects in different tissues may vary.

In this report, we unexpectedly found that the ER is the critical target of *Vcp* deficiency in regulating dendritic spine density in neurons. Our data suggest that VCP, P47 and ATL1 work together to control tubular ER formation and influence protein synthesis. Previous studies have suggested that the VCP/P47 complex regulates the activity of an unknown membrane fusion regulator, thereby controlling tubular ER formation^53^. Based on our data, it seems possible that ATL1 is the membrane fusion regulator of the VCP-P47 complex that regulates ER membrane fusion. In our TEM analysis, we found that the length of rER, ribosomal attachment on rER and rER abundance were noticeably reduced in VCP R95G knockin neurons. Although rER is generally believed to contribute to synthesis of membrane/secreted proteins, several studies have demonstrated that a noticeable fraction of cytosolic proteins are also synthesized by ribosomes associated with ER^33^,^34^,^35^. Thus, ER defects caused by *Vcp* deficiency likely results in a protein synthesis deficit. Using 1-h AHA labeling, we show here that protein synthesis efficiency is impaired by a *Vcp* deficiency, which is consistent with the ER defects revealed by TEM. Further, expression of synaptic glutamate receptors GRIN2B and GRIA2/3—the important ion channels of excitatory synapses—was reduced in VCP R95G knockin mutant neurons, indicative of VCP involvement in the impairment of the synaptic response and consequent elimination of dendritic spines. It would be intriguing to explore in the future whether the VCP R95G mutation results in impairment of electrophysiological responses and cognitive deficiencies using our knockin mutant mice.

In our experiments, we did find an unfolded protein response induced by *Ufd1l* knockdown and tunicamycin treatment. However, the dendritic spine density was not affected by *Ufd1l* knockdown or tunicamycin treatment, suggesting that an unfolded protein response is not involved in regulation of dendritic spine density. It is noteworthy that protein translation was transiently suppressed by tunicamycin treatment in neurons. Perhaps protein translation suppression is too transient to noticeably influence the total amounts of synaptic membrane proteins, thereby explaining the lack of effect on dendritic spines. Although an unfolded protein response, either induced by *Ufd1l* knockdown or tunicamycin treatment, seems not to be critical for regulation of dendritic spine density under our experimental conditions, it is still possible that an unfolded protein response influences other neuronal functions. This would be a topic worth pursuing in the future. In addition to protein degradation, the VCP–UFD1L–NPL4 complex also regulates other activities, such as the ubiquitin-governed DNA damage response^54^. As DNA double-stranded breaks are involved in expression of neuronal early-response genes^55^, it would also be intriguing to investigate whether VCP contributes to regulation of neuronal early response genes. It may not directly control dendritic spine formation but likely regulates other neuronal functions.

In this report, we provide evidence that changes in ER structure and function may be a common characteristic of various neurological disorders, including IBMPFD and SPG3A. SPG3A is characterized by progressive motor weakness and spasticity^56^. Degeneration of corticospinal tract axons with the onset of childhood is the major phenotypic feature of SPG3A. In addition, recent studies have also indicated hypometabolism in the frontal cortex and cerebellum and functional disorders in the frontal cortex in patients with SPG3A^57^,^58^. Our data indicate that expression of the *ATL1* mutant reduces the efficiencies of protein synthesis and dendritic spine formation in cultured hippocampal neurons. It seems likely that protein synthesis is also involved in the pathogenesis of SPG3A.

Branched-chain amino acids, particularly leucine, have been shown to bind sestrin2 to activate protein synthesis through the activation of the mTOR pathway in a variety of cells, including neurons^42^,^59^. Our data show that restoring protein synthesis using a leucine supplement efficiently increases the activity of S6 kinase, protein synthesis and the dendritic spine density of neurons. In contrast, a recent study indicated that leucine is a less efficient mTOR activator in *Vcp*-deficient muscle cells^60^. This is another example of a preferential effect of VCP on different cell types. Based on our observations, neurons are susceptible to leucine supplements, so taking a leucine supplement may be a good way of increasing protein synthesis in the brain.

In conclusion, our present study reveals that ER morphogenesis and protein synthesis play roles in dendritic spine formation. VCP, P47 and ATL1 regulate tubular ER formation, influence protein synthesis and then control dendritic spine formation. In light of these findings, we propose that, in addition to unfolded protein accumulation and aggregation, the role of ER morphology and protein synthesis in controlling protein homeostasis should be evaluated in neurological disorders. Restoring protein synthesis with leucine provides a potential therapeutic strategy.

## Methods

### Antibodies and chemicals

The antibodies and reagents used in this study were as follows: eIF2*α* (ref. 61) (sc-133,132, mouse, 1:1,000), Santa Cruz Biotechnology; VCP (612,182, mouse, 1:1,000), BD Transduction Laboratories; AHA (C10102), reaction buffer kits (C10269 and C10276), detection reagent Alexa Fluor 488 (A10267), GFP (A6455, rabbit, 1:1,000), Invitrogen; TAMRA (MA1-041, mouse, 1:1,000), P47 (PA5-21633, rabbit, 1:500), Calreticulin (PA3-900, rabbit, 1:200) and phospho-eIF2*α* (MA5-15133, rabbit, 1:1,000), Thermo; HA (3F10, rat, 1:500), Roche; MYC (9B11, mouse, 1:1,000), Phospho-S6 ribosomal protein (4856, rabbit, 1:1,000), S6 ribosomal protein (2217, rabbit, 1:1,000), Cell Signaling; GFP (ref. 62) (ab13970, chicken, 1:5,000), Abcam; puromycin (12D10, mouse, 1:1,000), GRIN2A (NR2A, 06-313, rabbit, 1:1,000) (ref. 63), GRIN2B (NR2B, 06-600, rabbit, 1:1,000) (ref. 63), GRIA1 (GluR1, MAB2263, mouse, 1:1,000), GRIA2/3 (GluR2/3, 07-598, rabbit, 1:1,000), GRM5 (mGluR5, AB5675, rabbit, 1:1,000), PSD-95 (MABN68, mouse, 1:2,000), Millipore; beta-actin (AC-74, mouse, 1:5,000), Sigma-Aldrich; CASK (mouse, 1:500) (ref. 64), rabbit polyclonal UFD1L (generated by immunization with full-length mouse UFD1L protein, 1:1,000); L-leucine, tunicamycin, cycloheximide and CGP57380 (Sigma-Aldrich); Rapamycin (LC Laboratories). The antibodies with validation profiles in Antibodypedia or 1DegreeBio are underlined.

### Plasmids

*Vcp* expression and knockdown plasmids have been described^28^. For the *Vcp* knockdown experiment, a plasmid cDNA6.2-GW/EmGFP-neg control, which expresses a miRNA that was predicted to not target any gene in mammalian genomes, was used as the non-silencing control in the knockdown experiments. Ub^G76V^-GFPref. 65 was obtained from Addgene (plasmid number 11941). Rat *P47* was amplified by RT-PCR with a pair of primers: forward, 5′- agatctGCGGAGGAGCGGCAGGACGCG -3′; backward, 5′- agatctTTATGTTAACCGCTGCACGA -3′. The PCR product was subcloned into BglII-linearized HA-Gw1-2b vector. Then, the *P47* fragment was further purified from HA-*P47* plasmid by KpnI and EcoRI and subcloned into KpnI and EcoRI-linearized Myc-Gw1-2b vector. For the *P47* knockdown construct P47i, a pair of oligonucleotides (5′- gatccccTGGTGACCTTAGAAGCTACttcaagagaGTAGCTTCTAAGGTCACCAttttta -3′ and 5′- agcttaaaaaTGGTGACCTTAGAAGCTACtctcttgaaGTAGCTTCTAAGGTCACCAggg -3′, the *P47* sequence is underlined) were annealed and subcloned into vector pSUPER.neo+GFP (SNG) (Oligoengine) according to the manufacturer's instructions. The vector SNG was then used as a non-silencing control for *P47* knockdown experiments. For the *P47* silent mutant that is resistant to P47i, the N- and C-terminal fragments P47-N and P47-C were individually amplified by PCR using the primer pairs: P47-N, SP6 and P47mut N primer 5′- TGGagatctCAGAAGCTAC -3′; P47-C, P47mut C primer 5′- GTAGCTTCTGagatctCCA -3′ and T7 primer (mutated nucleotides that did not influence the peptide sequence are underlined, lowercase characters indicate the BglII site). The PCR products were then digested with BglII/XmaI or SalI. Three-piece ligation was then performed to subclone the P47-N and P47-C fragments into XmaI-SalI linearized Gw1-Myc2b. Mouse Atl1 was amplified by PCR with a primer set: 5′- gaagatctATGGCTAAGAGCCGCAGGGA -3′ and 5′- acgcgtcgacTTAAATTTTCTTCTTTTCCG -3′. The recognition sites of BglII and SalI are underlined. The amplified products were subcloned into the Gw1-Myc2b vector. The R217Q mutant was generated by site directed mutagenesis with a primer set: 5′- CTGATATTTCTTGTTCaAGACTGGAGTTTCCCA -3′ and 5′- TGGGAAACTCCAGTCTtGAACAAGAAATATCAG -3′. The lowercase characters represent the single nucleotide mutation sites. Mouse Rab10 was amplified by PCR with a primer set: 5′- gaagatctATGGCGAAGAAGACGTACGA -3′ and 5′- acgcgtcgacTCAGCAGCACTTGCTCTTCC -3′. The underlined nucleotides represent the recognition sites of BglII and SalI. The amplified products were also subcloned into the Gw1-Myc2b vector. The T23N mutant was generated by site directed mutagenesis with the primer set: 5′- TCGGGAGTGGGCAAGAaCTGCGTCCTTTTTCGT -3′ and 5′- ACGAAAAAGGACGCAGtTCTTGCCCACTCCCGA -3′. The lowercase characters indicate the mutated residues. Ub-CD3δ-GFP was constructed by amplifying CD3δ by PCR from mouse spleen cDNA using primers 5′- cggggtaccATGGAACACAGCGGGATT -3′ and 3′- gcgggatccCAGATTTCTTGTTCCGGGG -5′, followed by subcloning into KpnI and BamHI linearized pEGFP-N2 vector. DsRed-ER (Cat. no. 6982-1) was purchased from Clontech. For in utero electroporation, *Vcp* knockdown and non-silencing control fragments were subcloned into pCAGIG (Addgene, no. 11159) vector at EcoRI site. The GFP fragment of the pCAG-GFP (Addgene, no. 11150) vector was removed by KpnI and NotI digestion, followed by Klenow fill-in and blunt end ligation. DsRed-ER fragments and full-length *P47* fragments were then individually subcloned into EcoRI linearized pCAG vector to generate pCAG-DsRed-ER and pCAG-myc-P47.

### Cell line

COS-1 cells were originally obtained from the Bioresource Collection and Research Center, Taiwan. The transfection protocol with Lipo2000 reagent (Invitrogen) was as described previously^28^. Briefly, a mixture containing 0.8 μg DNA and 4 μl Lipo2000 was added into each well of 12-well culture plate to deliver DNA into COS cells based on manufacture instruction.

### Animals

All animal experiments were performed with the approval of the Academia Sinica Institutional Animal Care and Utilization Committee and in strict accordance with its guidelines and those of the Council of Agriculture Guidebook for the Care and Use of Laboratory Animals. Animals were housed in the animal facility of the Institute of Molecular Biology, Academia Sinica, with a 14 h light/10 h dark cycle and controlled temperature and humidity. SD pregnant rats and C57BL/6 pregnant mice at embryonic day 18 (E18) were used to prepare cultured hippocampal and cortical neurons. For in utero electroporation, CD1 (ICR) pregnant mice at E15.5 were used. Electroporated pups regardless of gender were analyzed at postnatal day 3 (P3).

### Neuronal culture and analyses

The detailed procedures for preparation and calcium phosphate precipitation of rat and mouse hippocampal cultures and the indirect fluorescence immunostaining have been previously described^28^,^29^,^66^. Briefly, hippocampal neurons from E18.5 embryos were seeded on coverslips (18 mm in diameter and 0.12–0.17 mm in thickness) coated with poly-L-lysine. Calcium phosphate precipitation was carried out using Hepes buffered saline at pH 7.05–7.07. A total 2.5 μg DNA was used for two wells. Neurons were then fixed with phosphate buffered saline (PBS) containing 4% paraformaldehyde and 4% sucrose and permeabilized with PBS with 0.2% Triton-X-100. A PBS solution containing 3% bovine serum albumin was used for blocking and antibody incubation for immunofluorescence staining. Images of neurons were recorded with a confocal microscope (LSM700, Zeiss) equipped with a Plan-Apochromat 63 × NA 1.4 oil objective lens (Zeiss) and captured with Zen acquisition and analysis software (Zeiss) at 20–22 °C as a Z-series of 5–12 sections spaced 0.6–0.8 μm apart. The Z-series was then projected into single images. For publication, the images were processed with Photoshop (Adobe), with minimal adjustment of brightness to the whole images. To analyze dendritic spine density, the spine numbers of dendrite fragments of 20 μm in length starting from a point 20 μm away from the soma were manually counted using ImageJ software. As dendritic spine formation is highly sensitive to culture conditions, such as the quality of the B27 supplement^67^, each experiment was repeated using the same lot of culture medium. The data from independent experiments were pooled for statistical analysis only when the variation of the control group was not significantly different between repeated experiments. To minimize the effects of bias, the critical experiments were performed blind by relabeling the samples with the assistance of other lab members.

### ER distribution

Cultured neurons were cotransfected with DsRed-ER and the various plasmids as indicated in each experiment. Immunostaining with corresponding antibodies to monitor expression of transfected genes was then performed. Cell images were acquired using a confocal microscope (LSM700, Zeiss) equipped with a Plan-Apochromat 63 × NA 1.4 oil objective lens (Zeiss) and driven by Zen acquisition and analysis software (Zeiss) at 20–22 °C as a Z-series spaced 0.36 μm apart. The Z-series was then projected into a single image to quantify the ER intensities. Neuronal morphology was first outlined based on Myc tag (WT and R95G of VCP) or GFP (control vector, VCPi and P47i) signals using the ‘Segmented Line' tool of ImageJ software. The intensities of DsRed-ER in the entire cell and soma were then quantified using the ImageJ measurement tool. Total dendritic DsRed-ER signals were then obtained by subtracting the soma intensities from the whole cell intensities. The ratio of the dendritic signal to somatic signal indicates the dendritic distribution of the ER. To further analyze the ER distribution along dendrites, cumulative probability was also measured. Radius areas were plotted from the soma margin every 5 μm up to a distance of 60 μm away from the soma. The cumulative probability distribution of the ratio of each radius intensity to total dendritic intensities is shown. In addition to ImageJ, quantification was also conducted with the Imaris software package (BitPlane, MN, USA). For this, only one panel of triplicates was verified. The Z-series were processed with the ‘Surface Creation' function of Imaris for the DsRed-ER channel. The surface was created by the absolute intensity without filtering. To define the ER signals of the entire neuron, a threshold range (between 18–255 shades/levels of grey) was used to include ER signals in the somata and all dendritic branches. Soma intensities were then quantified by the ‘Segment of Interest Regions' tool to outline the soma area with the same threshold. Background surface intensities were manually deleted. The intensity results were then exported to excel for statistical analyses with GraphPad Prism. The representative images were outputted to a TIFF file by a ‘snapshot' of the scene. Images were then processed with photoshop for publication. For dsRed2-ER and calreticulin morphology in neurons, super-resolution microscopy and 3D-reconstruction was performed using an Elyra PS.1 microscope (Carl Zeiss) equipped with 63 × /NA 1.4 oil (Plan-Apochromat; Carl Zeiss) objective lens and iXon 885 EMCCD (Andor Technology) at room temperature. The Z-series spaced 0.13 μm apart was set and processed with Zen 2011 software: noise filter parameter value, -3; and SR frequency weighting value, +1.

### *In utero* electroporation and immunohistochemistry

Expression plasmids were delivered to ventricular radial glial cells by electroporation as described^68^. Briefly, at E15.5, pregnant ICR (CD1) mice purchased from BioLASCO (Taiwan) were anesthetized with a MATRX isoflurane VIP 3000 vaporizer (Midmark). One μg μl^−1^ of DNA plasmid mixture (with 0.01% fast-green dye) in 0.9% NaCl was then injected in the ventricles. Five pulses of 40 mV, 50 ms with 950 ms intervals were generated using Electro Square Porator ECM830 (BTX). Brain sections were then prepared from P3 pups regardless of gender for immunofluorescence staining as described^69^. Briefly, the procedure was similar to the method described in above ‘Neuronal Culture and Analysis', except that 30-μm-think sections and 3% normal horse serum in Tris-buffered saline for blocking and antibody incubation were used. Images were collected and analyzed as described above.

### VCP R95G knockin mice

A recombineering-based method was used to generate knockin mutant mice^70^. Briefly, a genomic fragment covering from intron 1 to intron 4 of the *Vcp* gene was subcloned from bacterial artificial chromosome clone RP23-343E15 into a modified pBluescript vector. The R95G point mutation at the third exon and an *Eco*RV site in the third intron were generated by direct mutagenesis. The *lox*P-flanked Neo cassette was then introduced into the third intron following the edited *Eco*RV site (Fig. 4a). The targeting vector was electroporated into C57BL/6 embryonic stem cells for production of chimeric mice. Breeding with C57BL/6-C2J albino strains was performed to accelerate germline transmission. Genotyping was examined by three methods: (1) Genomic Southern blotting was performed using a 5′ probe (nucleotide residues 106,884–107,133 of clone RP23-343E15) and 3′ probe (nucleotide residues nucleotide residues 106,884–107,133 of clone RP23-343E15), respectively. (2) Genomic PCR was carried out using primer set 1: 5′- GTGTCTGAAGACAGTGGACAGTGT -3′, 5′- GAAGAGCTTGGCGGCGAATG -3′ and 5′- CTTAGAAATGAGACCAGAACCGGG -3′ (Fig. 4a). (3) After removing the Neo cassette by breeding with E2A-Cre mice (stock no. 003724, Jackson lab), offspring were genotyped using primer set 2: 5′- TCAGTGACCCAAAGTCCTTAGC -3′ and GGGAGACGGTGTCTATAATGCAGA -3′. Since the VCP R95G allele contains an edited *Eco*RV site, the PCR amplified product was further processed with *Eco*RV digestion (FastDigest, Thermo Scientific) to generate two fragments of comparable size. Sequencing was performed to confirm the specific mutated residues.

### Transmission electron microscopy

For transmission electron microscopy (TEM) study, cortical neurons were plated on Aclar embedding film (Electron Microscopy Sciences). At 10 DIV, cells were fixed with fixative buffer (2.5% glutaraldehyde in 0.1 M sodium cacodylate, pH 7.4) for 1 h at 4 °C. After washing with washing buffer (0.1 M sodium cacodylate (Sigma), 4% sucrose, 0.05% CaCl_2_) for 5 min, cells were post-fixed with 1% OsO_4_ (Electron Microscopy Sciences) in 0.1 M sodium cacodylate for 1 h at 4 °C, washed with cold ddH_2_O, for 5 min three times, and incubated in 1% uranyl acid (Polysciences) for 1 h at 4 °C. The samples were then dehydrated with graded ethanol solutions at RT for 7 min for each step: 50% once, 70% once, 90% once and 100% three times. Then, cells were filtrated with a series of solutions as follows: (1) EtOH:Spurr's Resin (Low Viscosity Embedding Media Spurr's, Kit Electron Microscopy Sciences, Hatfield, PA)=1:1 for 30 min; (2) EtOH:Spurr's Resin=1:2 for 40 min; (3) pure Spurr's Resin for 1 h. Cells were then polymerized at 70 °C for 20 h. Ultrathin sections were sectioned with a diamond knife (DiATOME) and stained with 4% uranyl acid for 5 min followed by lead citrate stain for 8 min. After washing with ddH_2_O, cells were examined by TEM (Tecnai G2 Spirit TWIN, FEI Company) with a Gatan CCD Camera (794.10.BP2 MultiScanTM) and acquisition software DigitalMicrograph (Gatan).

### *De novo* protein synthesis monitored by BONCAT and SUnSET

For BONCAT^30^, cells were washed with PBS once, then incubated with Met-free DMEM (GIBCO, Invitrogen) for 30 min. L-azidohomoalanine (AHA, 50 μM) was added into the medium for incubation for another 1 h. Cells were then washed with PBS three times and fixed with 4% paraformaldehyde for 15 min. Permeabilization was then performed with 0.2% Triton X-100 for 10 min. After washing with PBS 3 times and blocking with 3% BSA, cells were processed with Click-iT Cell Reaction Buffer Kit (Invitrogen) according to the manufacturer's instructions. Briefly, cells were incubated with Click-iT reaction buffer (5 μM alkyne modified Alexa Fluor-488) for 30 min. To identify transfected neurons, cells were further washed with PBS three times, blocked with 3% BSA again and subjected to standard fluorescence immunostaining with Myc antibody and Alexa Flour-555-conjugated secondary antibody. Alexa Fluor-488 intensities (indicative of AHA incorporation) in the soma and dendrites (a 20 μm fragment 20 μm apart from the soma of transfected neurons) were analyzed using ImageJ. Some of the critical experiments were performed blind to minimize the effects of bias.

For SUnSET^36^, cells were transferred to neuronal medium containing puromycin (10 μg ml^−1^) for 10 min. Cells were then washed with PBS three times and returned to the original medium for another 50 min incubation. Fixation and permeabilization were then performed as described above.

### Immunoprecipitation

COS-1 cells were transfected with myc-tagged Atl1 and HA-tagged *Vcp* for 24 h. Cells were harvested with lysis buffer (50 mM Tris, pH7.4 containing 1% Triton X-100, 2 μg ml^−1^ leupeptin, 2 μg ml^−1^ aprotinin, 10 μg ml^−1^ pepstatin, and 2 mM PMSF, 2 μM MG132) and centrifuged at 12,000 rpm at 4 °C. The solubilized extract was subjected to immunoprecipitation using Myc antibodies and control non-immune IgG combined with protein A sepharose. After mixing by rotation for 4 h at 4 °C, the precipitates were sequentially washed with the following solutions buffered with 10 mM Tris-HCl (pH7.4): 1% Triton X-100, once; 0.1% Triton X-100 and 0.5 M LiCl, twice; 0.5 M LiCl, once; and Tris buffer alone, once. The precipitated proteins were then analyzed by immunoblotting.

### Immunoblotting

To detect phosphorylated proteins, sodium fluoride (50 mM), sodium orthovanadate (1 mM) and sodium butyrate (1 mM) were included in lysis buffer (2% SDS in 50 mM Tris-HCl, pH 7.5, 150 mM NaCl, 1 mM EDTA, 1 mM EGTA, 2 μg ml^−1^ aprotinin, 2 mM PMSF, 2 μg ml^−1^ leupeptin, 2 μg ml^−1^ pepstatin, 2 μM MG132, 250 U of Benzonase endonuclease) to lyse cells. Proteins were separated by SDS-PAGE and blotted on PVDF membranes. The membranes were blocked with 5% nonfat milk or 3% BSA in TBS-T (50 mM Tris, pH7.4, 150 mM NaCl with 0.2% Tween 20) for 1 h at RT and incubated with indicated antibodies in 3% blocking solution for 2 h. After washing with TBS-T buffer three times, membranes were incubated with HRP-conjugated secondary antibodies for 1 h. For AHA incorporation, after washing with PBS three times, cells were harvested with lysis buffer (1% SDS in 50 mM Tris-HCl, pH 8.0 buffer with protease inhibitors and endonuclease as described above). Based on the Click-iT Protein Reaction Buffer Kit protocol (Invitrogen), each 100 μg of protein samples was then labeled with 40 μM alkyne modified tetramethylrhodamine (TAMRA). Protein samples were then subjected to immunoblotting as described above, except that anti-TAMRA antibody was used as primary antibody to detect AHA-labeled proteins. For SUnSET, anti-puromycin antibody was used. The antibody-bound complexes were detected with WesternBright Sirius HRP substrate (Advansta). The protein signals were then visualized and analyzed using ImageQuant LAS 4,000 with the software ImageQuant LAS 4,000 Biomolecular Imager (GE Healthcare). Images have been cropped for presentation. Full-size images are presented in Supplementary Fig. 9.

### Statistical analysis

All the quantitative data in this report are presented as means plus s.e.m. or cumulative distribution. Graphs were plotted using GraphPad Prism 5.0 (GraphPad software). No statistical method was applied to evaluate the sample size, but our sample sizes are similar to a previous publication^28^,^68^,^69^. Basically, 20–30 neurons were collected each time from three independent experiments. For dendritic spine analysis, three dendrites of each neuron were quantitated. Data collection and analysis were conducted randomly and blind. Most of the data meet the assumption of the tests (normal distribution), except the dendritic spine density. Statistical analysis was performed using the two-tailed unpaired Student *t*-test for two group comparisons. For three group comparisons, one-way analysis of variance with Bonferroni's test in GraphPad Prism 5.0 was used. Two-way ANOVA with Bonferroni's test using SigmaStat 3.5 was performed to analyze the effects of two genetic factors combined with two different treatments. For cumulative probability distributions of spine density, the statistical significance was analyzed with a Kolmogorov-Smirnov test (SPSS software, version 10.0, SPSS, Chicago, Ill). *P* values <0.05 were considered significant.

## Additional information

**How to cite this article:** Shih, Y.-T. and Hsueh, Y-P., VCP and ATL1 regulate endoplasmic reticulum and protein synthesis for dendritic spine formation. *Nat. Commun.* 7:11020 doi: 10.1038/ncomms11020 (2016).

## Supplementary Material

Supplementary Information Supplementary Figures 1-9
