# Supplementary material for: VCP and ATL1 regulate endoplasmic reticulum and protein synthesis for dendritic spine formation
Source: Nat Commun. 2016 Mar 17;7:11020. doi: 10.1038/ncomms11020 (PMC4800434; doi:10.1038/ncomms11020)
Supplement: Supplementary Information — Supplementary Figures 1-9 [file ncomms11020-s1.pdf]

Supplementary Information

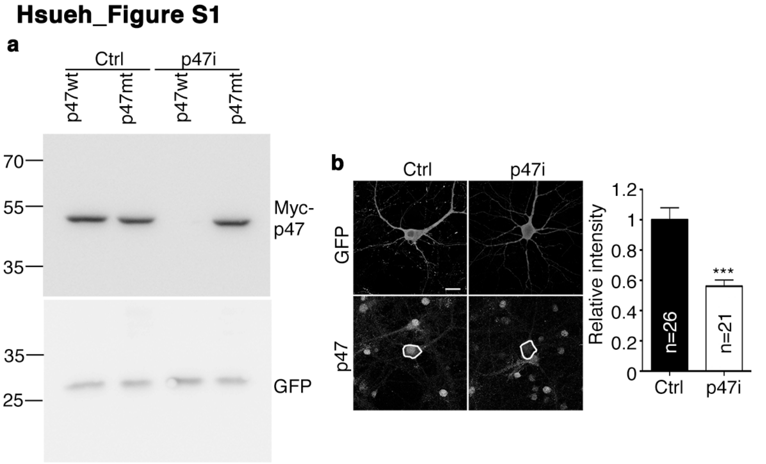

Supplementary Figure 1. The *p47* shRNA knockdown construct p47i reduces the protein levels of both overexpressed and endogenous p47. (**a**) The effect of the *p47* knockdown construct p47i on the expression of Myc-tagged WT p47 (p47wt) and the p47i-resistant silent mutant (p47mt) in transfected COS-1 cells. The shRNA expression vector Super.GFP+neo was used as a control. The representative images of three independent experiments are shown. (**b**) Endogenous *p47* in cultured hippocampal neurons is also downregulated by p47i. The relative p47 protein levels in the somata were quantified. The data from three independent experiments were analyzed. The means plus s.e.m. are presented. The sample sizes (n) of the examined neurons are indicated. ***, $P < 0.001$; unpaired t-test. Scale bar: 20 μm.

Hsueh_Figure S2

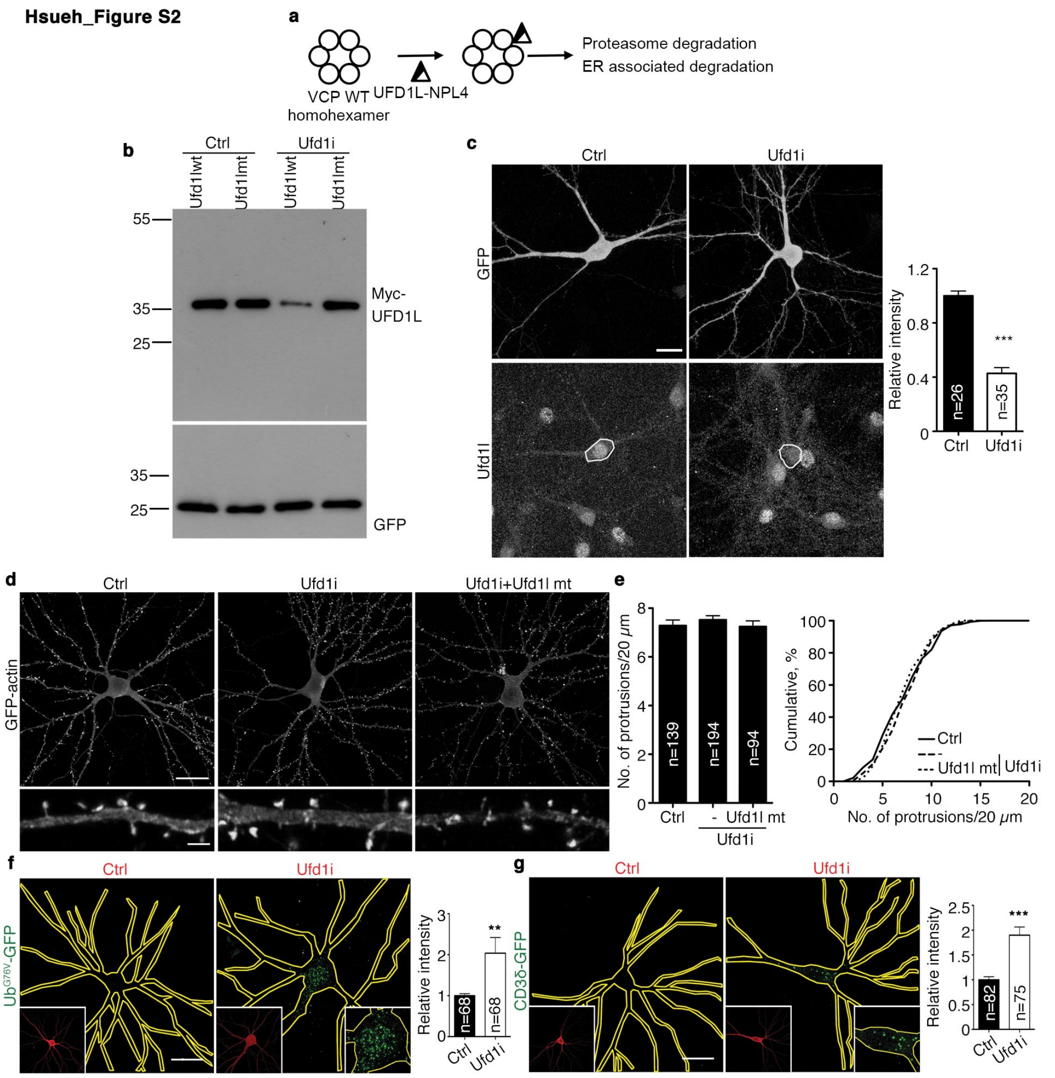

Supplementary Figure 2. *Ufd1l* knockdown does not reduce the density of dendritic spines. (**a**) A schematic of how the VCP hexamer and its cofactor, the UFDL1-NPL4 dimer, regulate protein degradation. (**b**) The effect of the Ufd1i RNA knockdown construct on the expression of Myc-tagged WT *Ufd1l* (Ufd1lwt) and the Ufd1i-resistant silent mutant (Ufd1lmt) in transfected COS-1 cells. The cells were harvested 24 h after transfection for immunoblotting with the indicated antibodies. The representative images of three independent experiments are shown. (**c**) The endogenous UFD1L levels in rat cultured hippocampal neurons are reduced by Ufd1i. The relative UFD1L protein levels in the somata were quantified by immunostaining. (**d**) UFD1L does not regulate dendritic spine density. Cultured rat hippocampal neurons were cotransfected with GFP-actin and the indicated plasmids at 12 days in vitro (DIV) and fixed for immunostaining at 18 DIV. Only the GFP signals that reveal the neuronal morphology are shown. The lower panels are enlargements of a segment of the dendrite for quantification. (**e**) Quantitation of protrusion densities in (**d**). The means plus s.e.m. and the cumulative probability distributions are shown. (**f**,**g**) Effect of *Ufd1l* knockdown on protein accumulation. Neurons were cotransfected with the non-silencing control or Ufd1i (coexpressing mCherry) and protein degradation reporter (**f**) Ub^{G76V}-GFP and (**g**) CD3δ-GFP, as indicated. The relative GFP intensities are shown. Transfected neurons are outlined in yellow, based on the mCherry signals shown in the left corner of each panel. The insets in the right corners of the panels are the enlarged images of the somata of the transfected neurons, which show GFP accumulation. Ctrl, non-silencing control. The data of the means plus s.e.m. from three independent experiments were analyzed. The sample sizes (n) of the analyzed neurons are indicated. Scale bar: original, 20 μm; enlarged, 2 μm. **, $P < 0.01$; ***, $P < 0.001$. Unpaired t-test (**c**,**f**,**g**); one-way ANOVA and Kolmogorov-Smirnov test for cumulative probability (**e**).

Hsueh_Figure S3

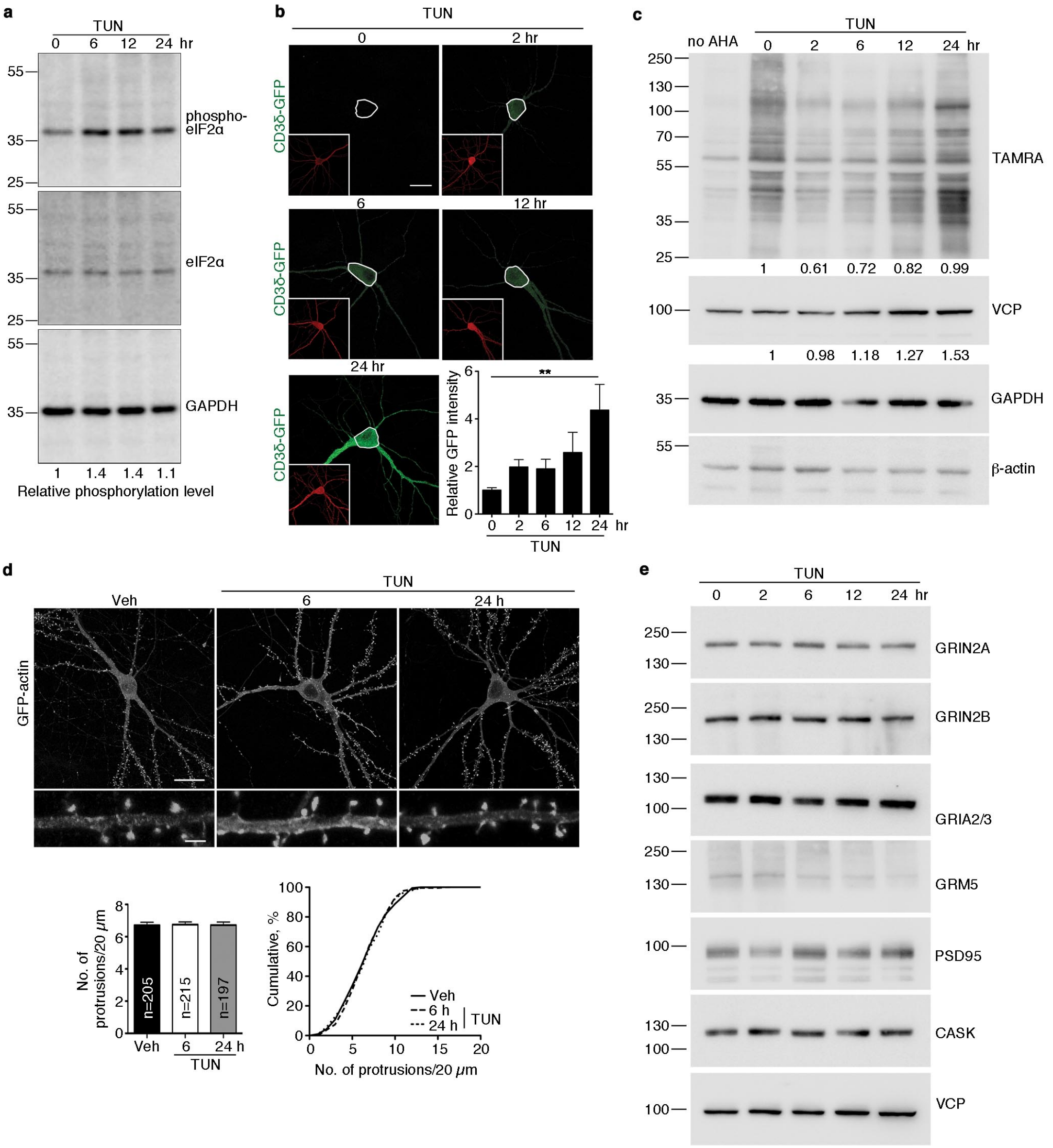

Supplementary Figure 3. Tunicamycin treatment has no effect on the dendritic spine density or expression of synaptic proteins. (**a**) Treatment with tunicamycin (TUN, 1 µg/ml) induces eIF2α phosphorylation in cultured neurons. (**b**) Tunicamycin treatment results in accumulation of unfolded protein reporter CD3δ-GFP in neurons in a time course-dependent manner. CD3δ-GFP and mCherry were cotransfected into hippocampal neurons at 12 DIV. Cells were harvested at 18 DIV to examine the expression of mCherry and CD3δ-GFP. The representative images and quantification of CD3δ-GFP are shown. (**c**) Tunicamycin treatment transiently suppresses protein translation of cultured hippocampal neurons, as revealed by AHA incorporation. AHA-labeled proteins were detected by TAMRA-conjugated alkyne and TAMRA antibody. Total TAMRA intensities were normalized to β-actin. (**d**) The induction of the ER stress response with TUN does not influence dendritic spine density. Cultured neurons were transfected with GFP-actin at 12 DIV and harvested for dendritic spine analysis at 18 DIV. TUN was added into cultures 6 and 24 hr before harvest. Representative images of GFP-actin and the quantitation of the protrusion densities and the cumulative probability distributions of neurons treated with TUN are shown. Veh, vehicle. The data of the means plus s.e.m. from three independent experiments were analyzed. The sample sizes (n) of the analyzed dendrites are indicated. Scale bars: original, 20 µm; enlarged, 2 µm. (**e**) Expression of synaptic proteins after tunicamycin treatment is not noticeably reduced. One-way ANOVA (**b**,**d**); Kolmogorov-Smirnov test for cumulative probability (**d**).

Hsueh_Figure S4

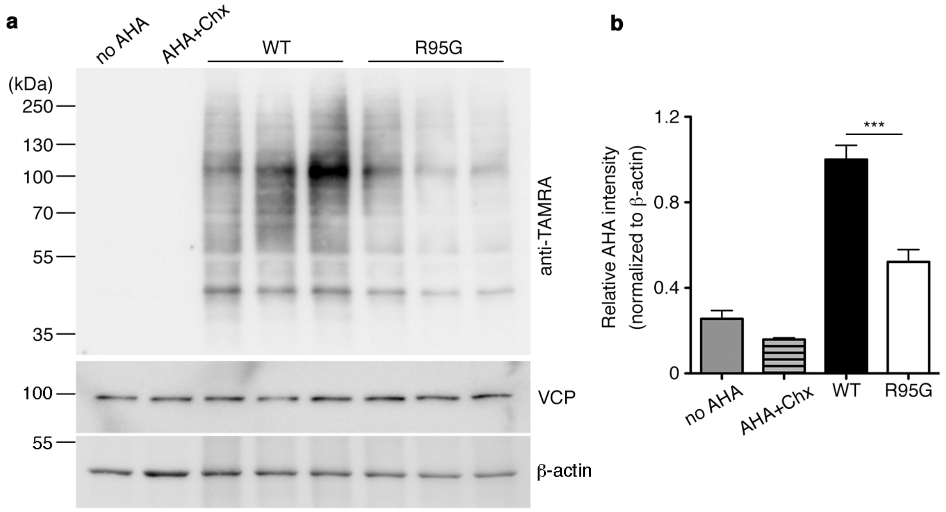

Supplementary Figure 4. VCP R95G knockin neurons also have a lower protein synthesis efficiency. (**a**) Newly synthesized proteins of cultured hippocampal neurons from VCP R95G knockin mice and wild-type littermates were labeled by AHA incorporation for 1 hr at 18 DIV. AHA-conjugated proteins were then detected by TAMRA-conjugated alkyne and TAMRA antibody. Cycloheximide (Chx) treatment blocked AHA labeling. (**b**) Quantification of total TAMRA intensities normalized to β-actin. The data of the means plus s.e.m. from six mice of each group were analyzed. ***, $P < 0.001$; unpaired t-test.

Hsueh_Figure S5

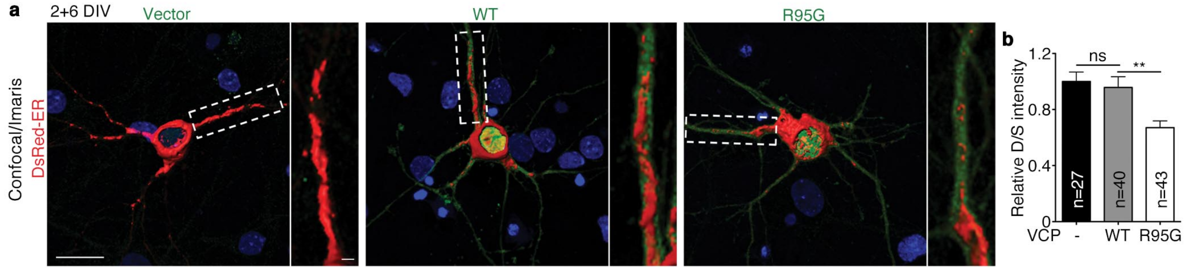

Supplementary Figure 5. Expression of the VCP R95G mutant impairs the dendritic ER distribution in immature neurons. At 2 DIV, cultured neurons were cotransfected with DsRed-ER and WT VCP or the R95G mutant, as indicated, and harvested for immunostaining at 8 DIV. (**a**) The representative IMARIS-processed images. (**b**) The D/S ratio of DsRed-ER. The data of the means plus s.e.m. from three independent experiments were analyzed. The sample sizes (n) of the analyzed neurons are indicated. ns, non-significant; **, $P < 0.01$; one-way ANOVA. Scale bars: original, 20 μm; enlarged, 2 μm.

Hsueh_Figure S6

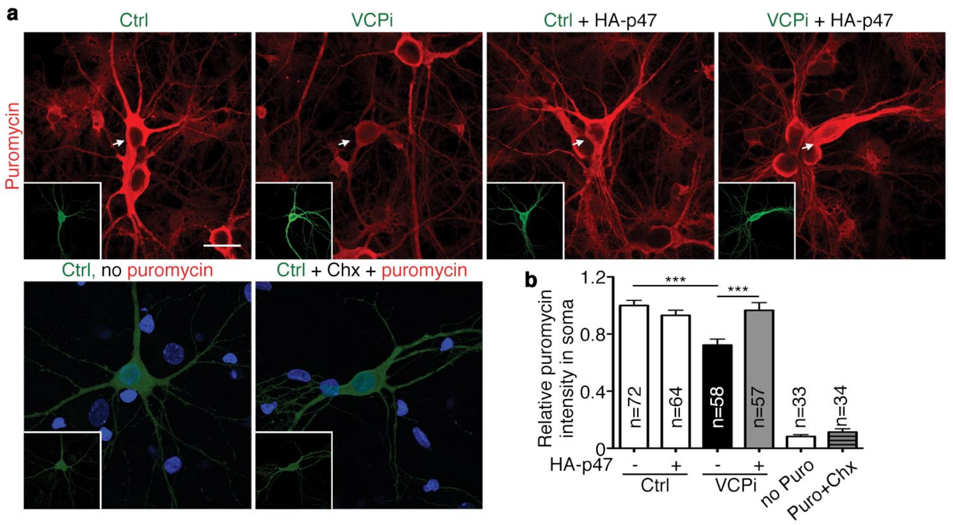

Supplementary Figure 6. SUnSET also reveals the deficits in protein synthesis in the *Vcp*-deficient neurons. Cultured neurons were cotransfected with the non-silencing control, the VCP knockdown construct and HA-tagged P47 at 12 DIV, as indicated. Puromycin (Puro) was then applied to the cultured neurons at 18 DIV to label newly-synthesized proteins. Immunostaining using GFP, HA and puromycin antibodies was then performed to analyze transfected neurons and protein synthesis. Cycloheximide (Chx) treatment blocked puromucin labeling. (**a**) The representative images. (**b**) The quantification of puromycin intensities in the somata. Ctrl, non-silencing control. The data of the means plus s.e.m. from three independent experiments were analyzed. The sample sizes (n) of the analyzed neurons are indicated. Scale bar: original, 25 µm. ***, $P < 0.001$; two-way ANOVA.

Hsueh_Figure S7

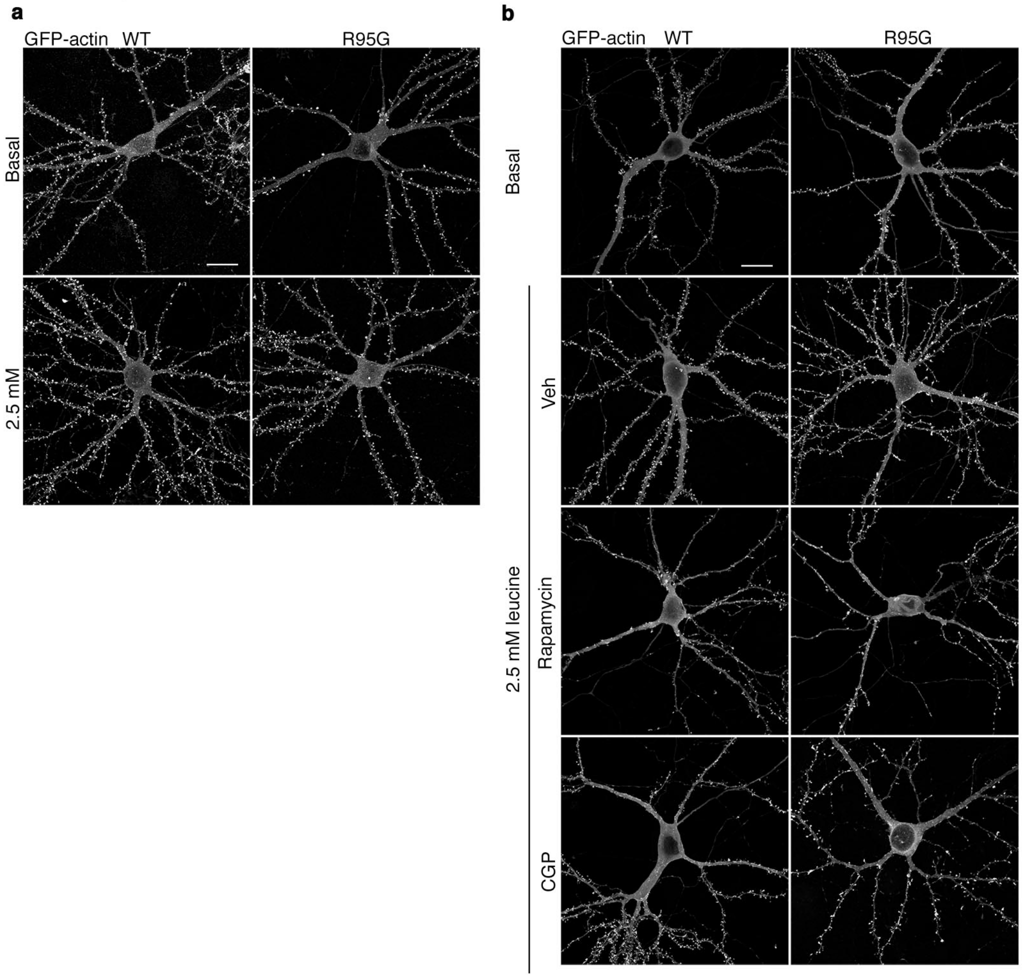

Supplementary Figure 7. Leucine rescues the defects of dendritic spine density caused by *Vcp* deficiency in neurons. (**a**) The original cell images of Figure 9f. (**b**) The original cell images of Figure 9g. Scale bars: 20 µm

Hsueh_Figure S8

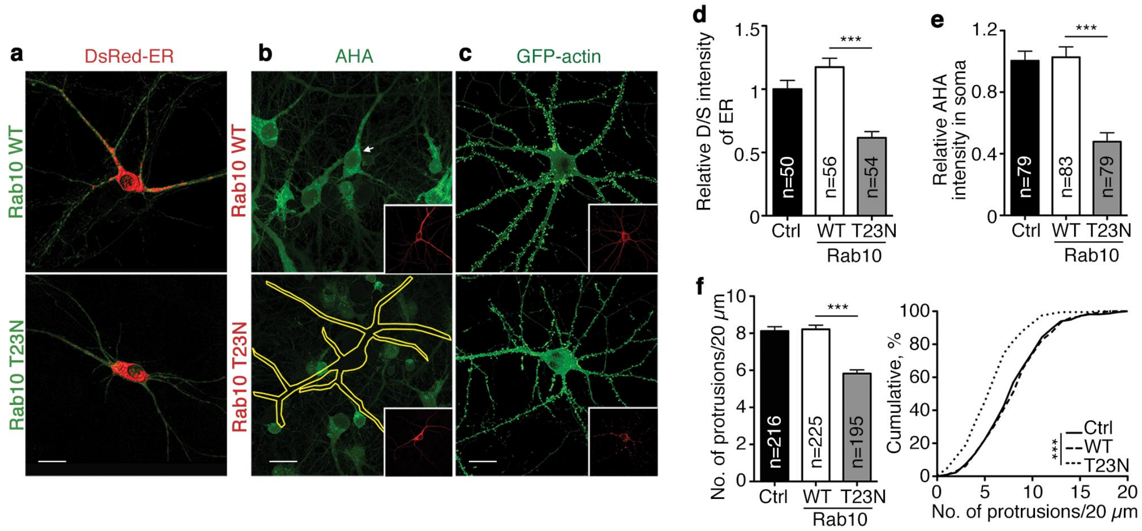

Supplementary Figure 8. RAB10 regulates protein synthesis and dendritic spine density. Rat cultured hippocampal neurons were transfected with plasmids, as indicated, and subjected to analyses of (**a,d**) ER morphology, (**b,e**) protein synthesis based on AHA labeling and (**c,f**) neuronal morphology using GFP signals. Transfected neurons are indicated either by arrowheads or yellow outline in (**b**). ER signals in (**a**) were processed with the Surpass Mode of the Imaris software (Bitplane). (**d**) Quantification of the ratio of dendritic ER to somatic ER. (**e**) Relative intensities of AHA-Alexa fluor 488 in soma. (**f**) Quantitation of dendritic protrusion densities. Scale bar: (**a**-**c**) 20 µm. Error bars indicate mean plus s.e.m.. Cumulative probability distributions of spine density are also shown in the right panel. The sample sizes (n) are indicated. ***, $P < 0.001$. One-way ANOVA (**d**,**e**,**f**); Kolmogorov-Smirnov test for cumulative probability (**f**). Note that the experiments were carried out at the same time as the experiments shown in Figure **10a-f**. Thus, the same vector control is used in Figure **10a-f**.

Hsueh_Figure S9

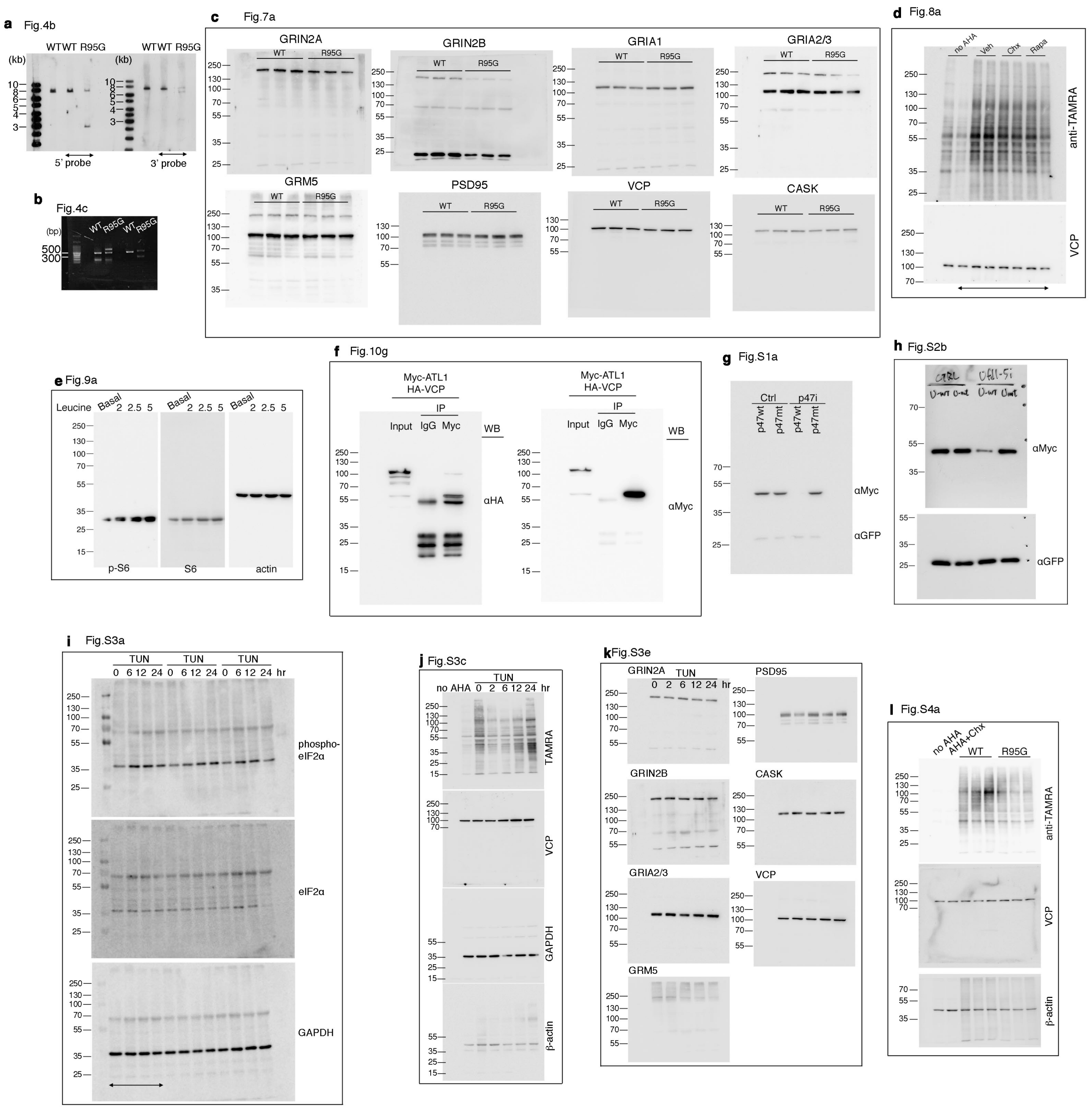

Supplementary Figure 9. Full blots for all experiments. Corresponding figures are indicated.
